# Supplementary material for: Sulfur defect engineering controls Li2S crystal orientation towards dendrite-free lithium metal batteries
Source: Nat Commun. 2025 Apr 1;16:3130. doi: 10.1038/s41467-025-57572-5 (PMC11962132; doi:10.1038/s41467-025-57572-5)
Supplement: Supplementary file 1 — Supplementary Information [file 41467_2025_57572_MOESM1_ESM.pdf]

Supplementary Information

Sulfur defect engineering controls Li_2S crystal orientation towards dendrite-free lithium metal batteries

Jin-Xia Lin[1,#], Peng Dai[1,#], Sheng-Nan Hu[1,#], Shi-Yuan Zhou[1], Gyeong-Su Park[2,3], Cheng-Guang Shi[1], Jun-Fei Shen[1], Yu-Xiang Xie[1], Wei-Chen Zheng[1], Hui Chen[1], Shi-Shi Liu[1], Hua-Yu Huang[1], Ying Zhong[1], Jun-Tao Li[4], Rena Oh[5*], Xiaoyang (Jerry) Huang[6], Wen-Feng Lin[7*], Ling Huang[1,8*], and Shi-Gang Sun[1,6,8*]

[1] State Key Laboratory of Physical Chemistry of Solid Surfaces, Collaborative Innovation Center of Chemistry for Energy Materials, College of Chemistry and Chemical Engineering, Xiamen University, Xiamen, China

[2] Institute of Next-Generation Semiconductor Convergence Technology, Daegu Gyeongbuk Institute of Science and Technology (DGIST), Daegu, Republic of Korea

[3] Department of Materials Science and Engineering and Research Institute of Advanced Materials, Seoul National University, Seoul, 08826, Republic of Korea

[4] College of Energy, Xiamen University, Xiamen, 361005, People's Republic of China

[5] School of Energy and Power Engineering, Chongqing University, Chongqing 400044, China

[6] Center of Advanced Electrochemical Energy, Institute of Advanced Interdisciplinary Studies, School of Chemistry and Chemical Engineering, Chongqing University, Chongqing 400044, China

[7] Department of Chemical Engineering, Loughborough University, Loughborough, Leicestershire, LE11 3TU, U.K.

[8] Innovation Research Institute in Advanced Electronic Chemicals of Quzhou, Zhejiang 324000, China

[#]These authors contributed equally: Jin-Xia Lin, Peng Dai, Sheng-Nan Hu

[*]Corresponding authors. E-mail: rena_oh@cqu.edu.cn; w.lin@lboro.ac.uk; huangl@xmu.edu.cn; sgsun@xmu.edu.cn.

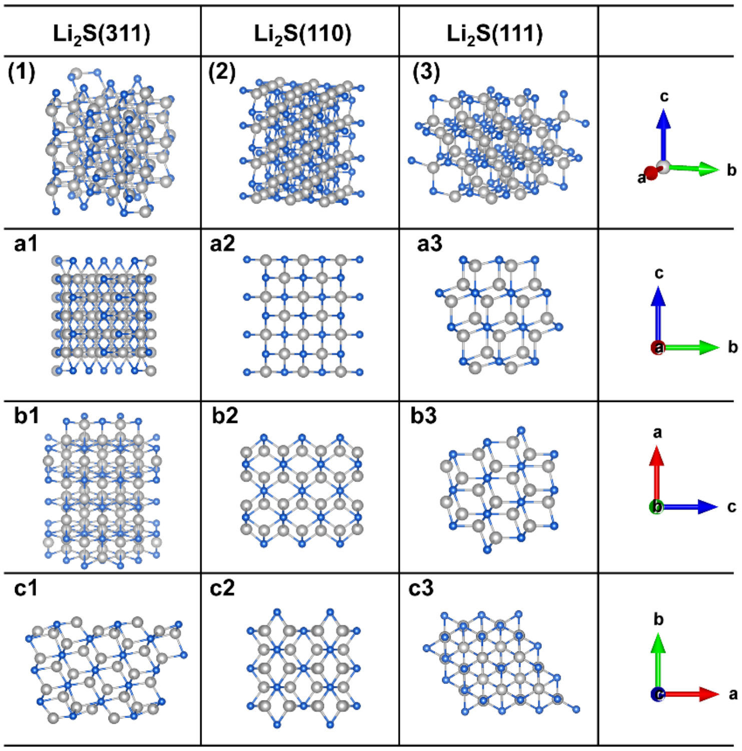

Supplementary Fig. 1| The optimized geometrical structure models of $Li_2S(311)$, $Li_2S(110)$ and $Li_2S(111)$. Gray: Li; Blue: S.

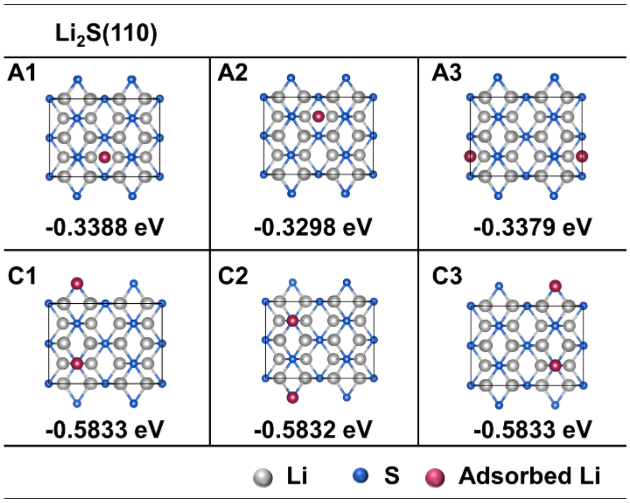

Supplementary Fig. 2| Schematics of Li conformations at different sites on $Li_2S(110)$. Gray: Li; Blue: S; Pink: adsorbed Li.

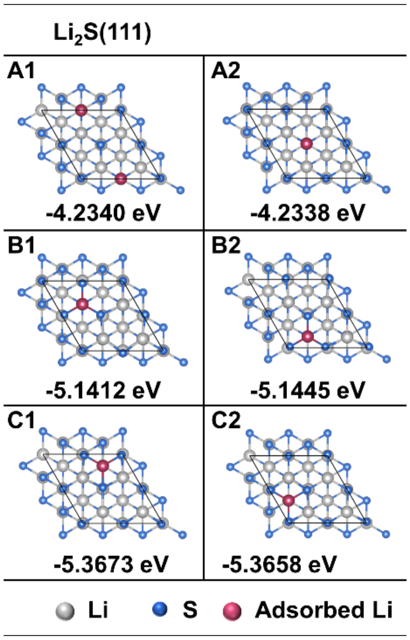

Supplementary Fig. 3| Schematics of Li conformations at different sites on $Li_2S(111)$. Gray: Li; Blue: S; Pink: adsorbed Li.

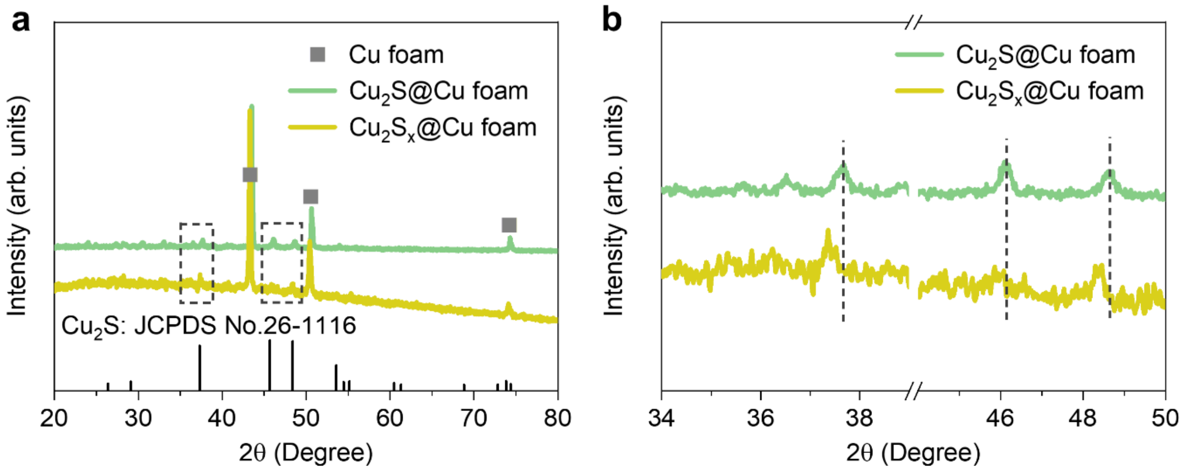

Supplementary Fig. 4| XRD patterns of Cu_2S@Cu and Cu_2S_x@Cu substrates. (a) XRD pattern. (b) The enlarged XRD pattern from the dashed box in supplementary Fig. 4a.

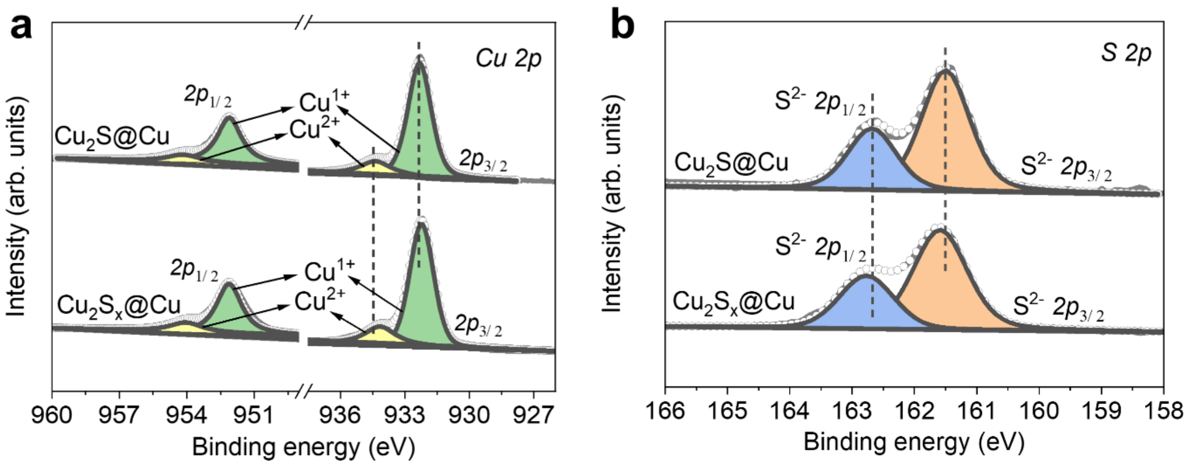

Supplementary Fig. 5| XPS survey spectra for Cu_2S@Cu and Cu_2S_x@Cu substrates. (a) *Cu 2p*. (b) *S 2p*.

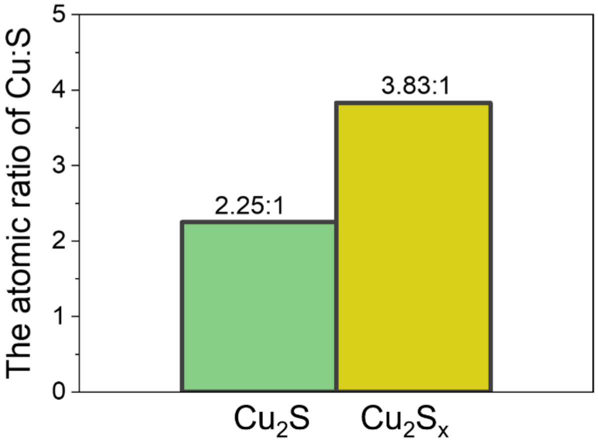

Supplementary Fig. 6| The atomic ratios of Cu:S from the XPS survey spectra for Cu_2S@Cu and Cu_2S_x@Cu substrates.

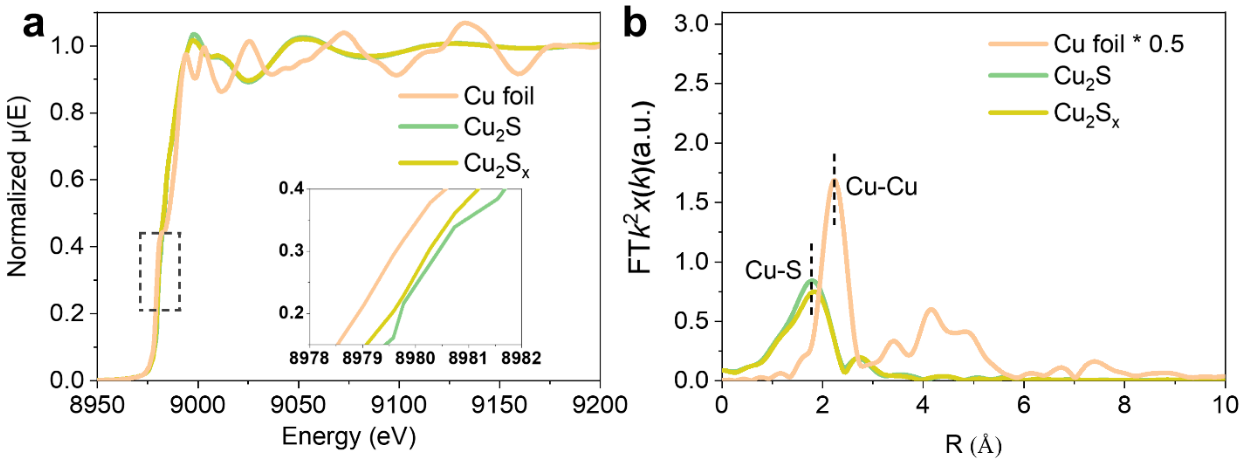

Supplementary Fig. 7| XAS analysis of Cu_2S and Cu_2S_x. (a) The Cu K-edge XANES spectra. (b) Fourier transforms of k^3-weighted EXAFS spectra.

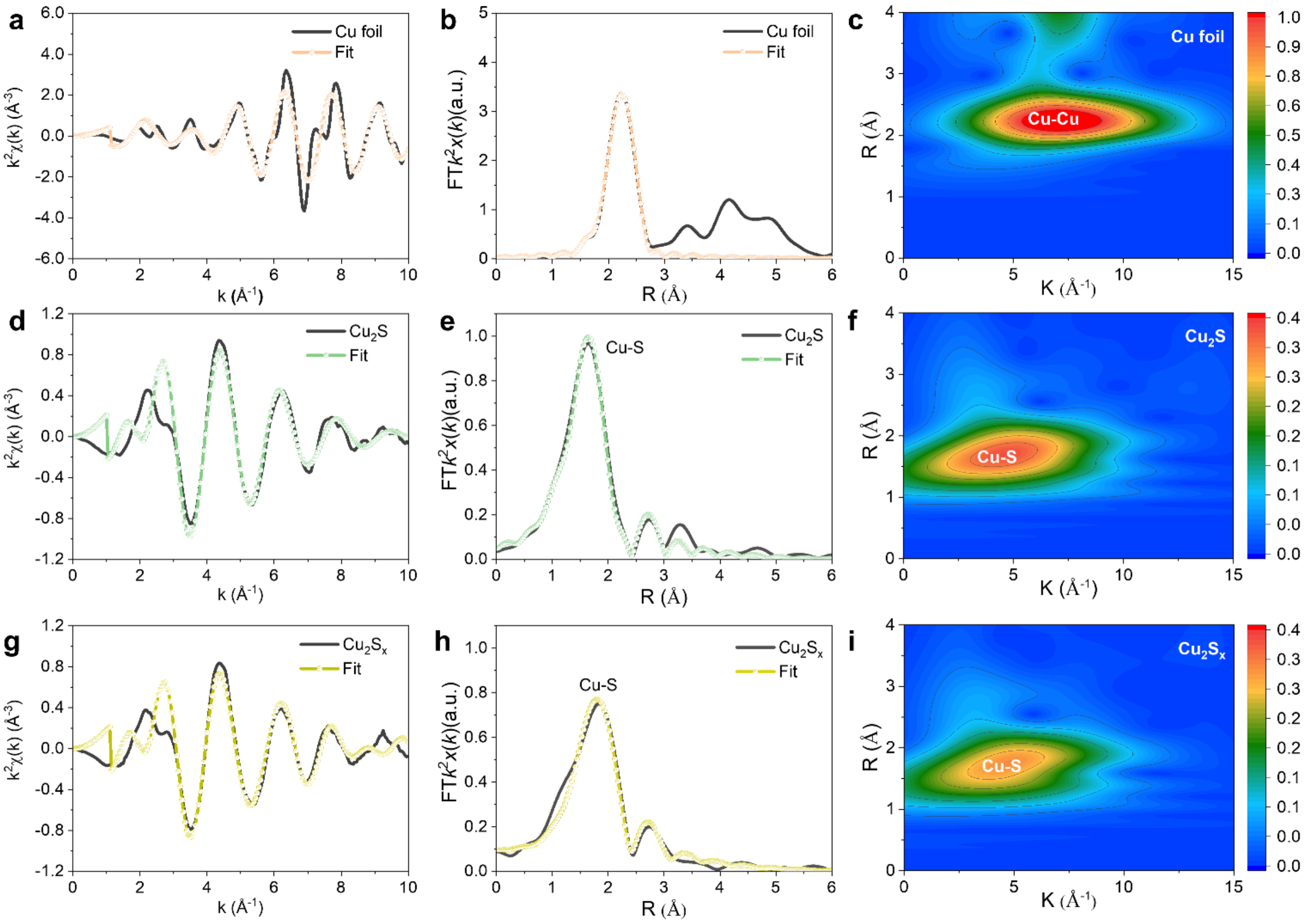

Supplementary Fig. 8| EXAFS analysis of Cu foil, Cu_2S and Cu_2S_x. (a, d, e) EXAFS oscillation extracted from K-edge spectra. (b, e, h) EXAFS in *R* spaces and (c, f, i) WT for the EXAFS signals of different samples: (a-c) Cu foil, (d-f) Cu_2S and (g-i) Cu_2S_x.

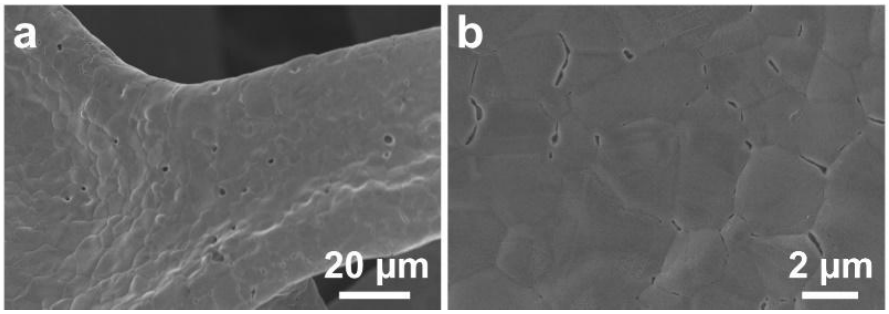

Supplementary Fig. 9| Morphological analysis of Cu foam. (a-b) SEM images.

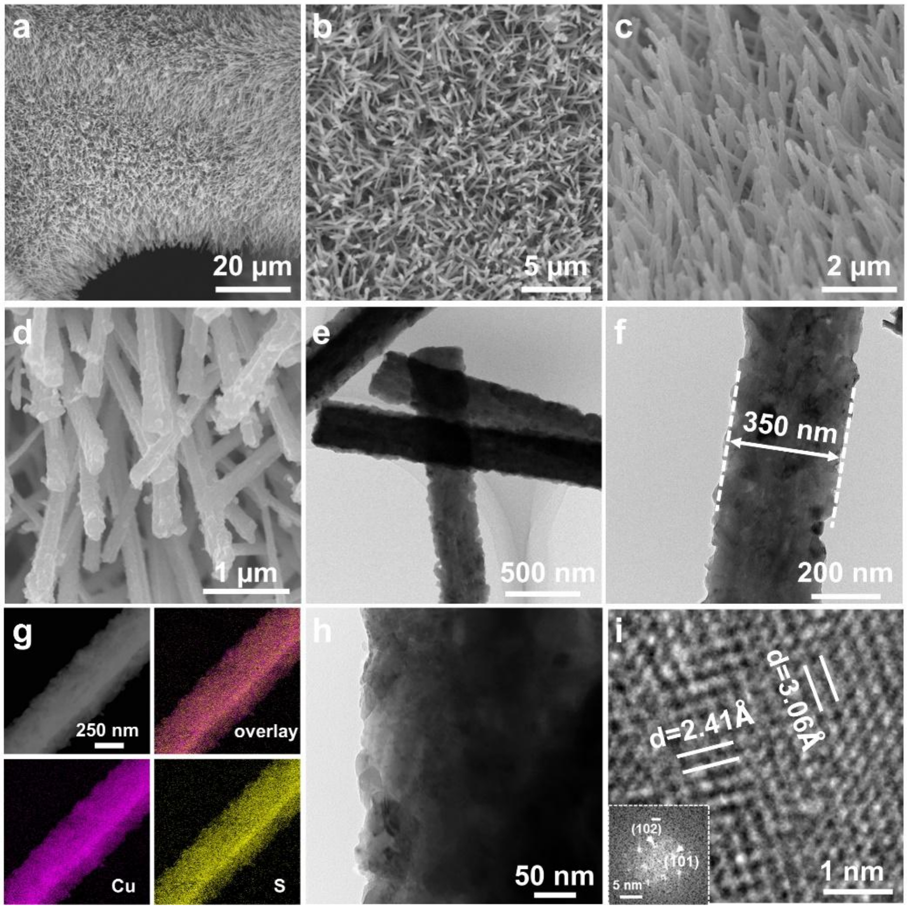

Supplementary Fig. 10| Morphological and structure analysis of Cu_2S@Cu NRs. (a-d) SEM images, (e-f) TEM images, (g) High-magnification elemental mapping, (h) TEM and (i) HRTEM image with the corresponding FFT diffractogram shown as an inset.

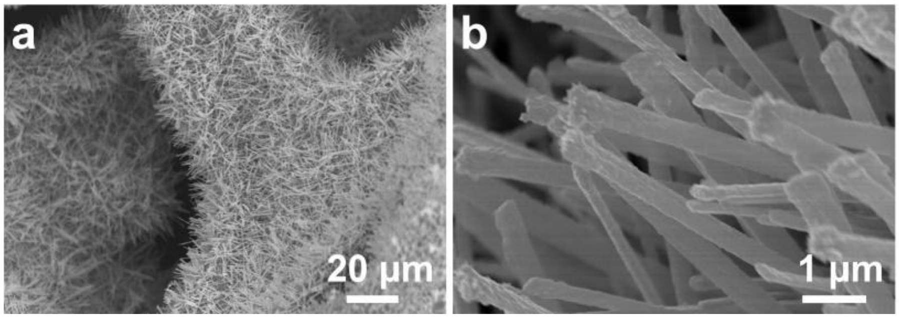

Supplementary Fig. 11| Morphological analysis of Cu_2S_x@Cu NRs. (a-b) SEM images.

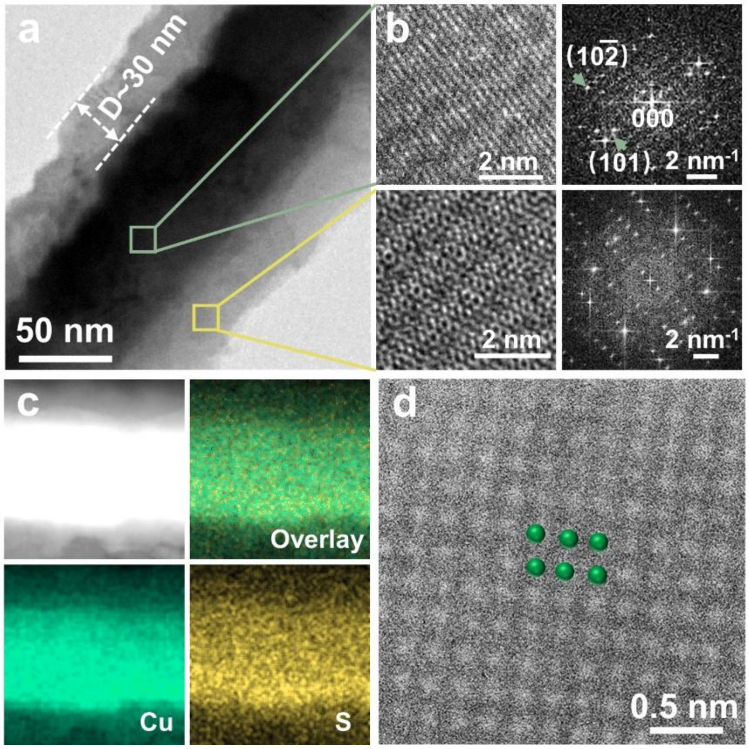

Supplementary Fig.12| Morphological and structure analysis of Cu_2S_x NRs. (a) TEM image. (b) The corresponding HRTEM image and FFT diffractograms of the selected area of the core (contains the (101) and (10$\bar{2}$) facet of *hexagonal*-Cu_2S) and the surface region (contains defective structures with satellite patterns). (c) High-magnification elemental mapping. (d) Atomic-resolution STEM image of the surface region with S defects exhibiting only Cu atoms.

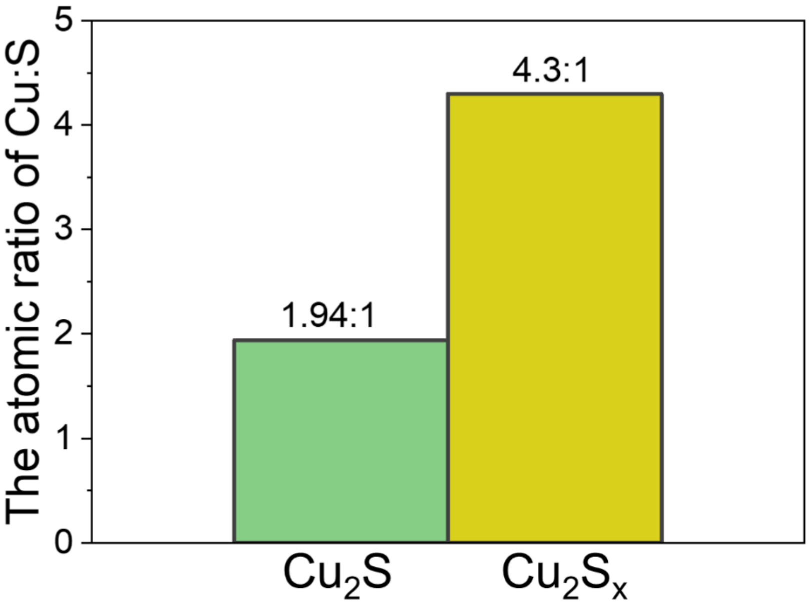

Supplementary Fig. 13| The atomic ratios of Cu:S from the EDS of Cu_2S@Cu and Cu_2S_x@Cu substrates.

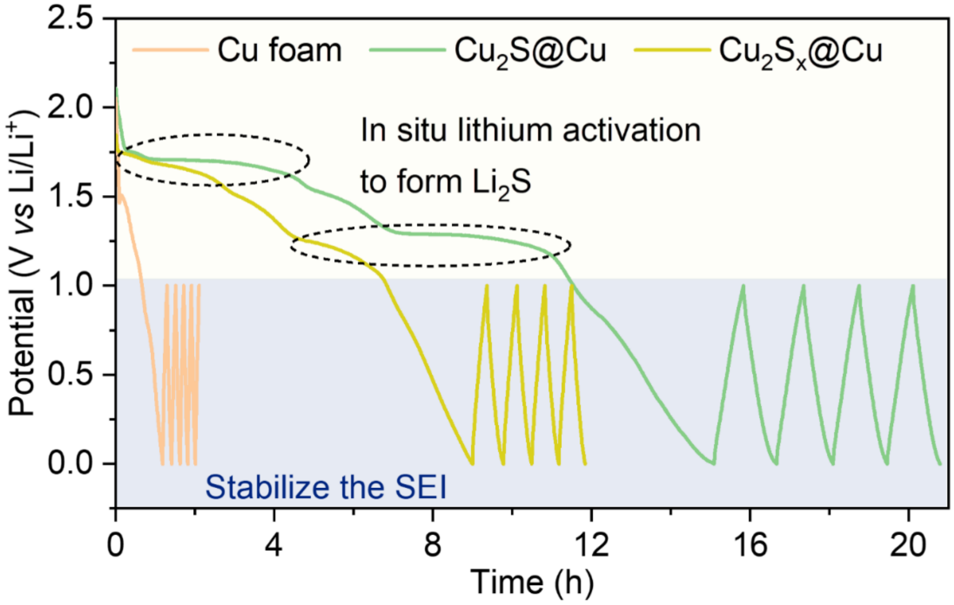

Supplementary Fig. 14| Galvanostatic discharge/charge (GDC) profiles of Cu foam, Cu_2S@Cu and Cu_2S_x@Cu electrodes between 0 and 1.0 V for 5 cycles. Applied current density: 0.05 mA cm^{-2}.

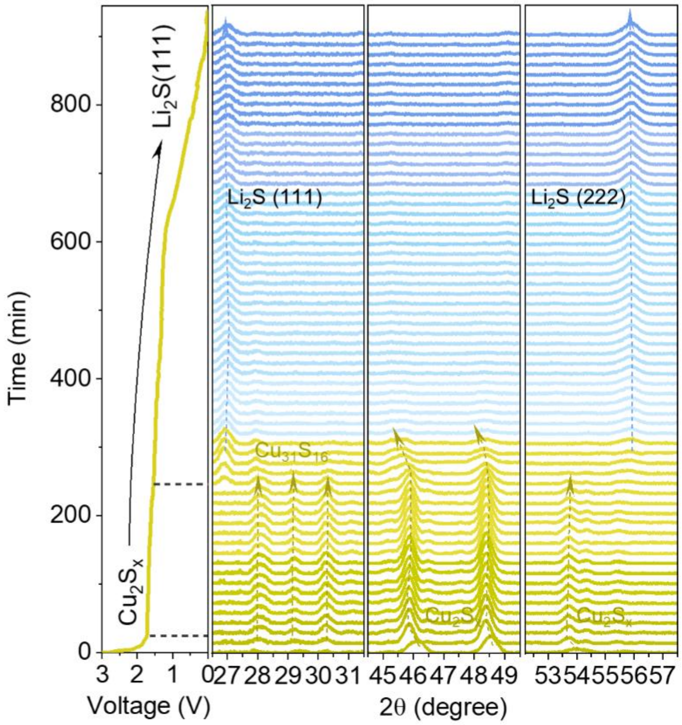

Supplementary Fig. 15| In situ XRD 1D maps and corresponding discharge curve of $Li_2S(111)$@Cu formation process. The dashed lines are the positions where the peaks shift occurs, the applied current density: 0.5 mA cm^{-2}, potential cutoff: 0.01 V, temperature: 25 ± 1°C.

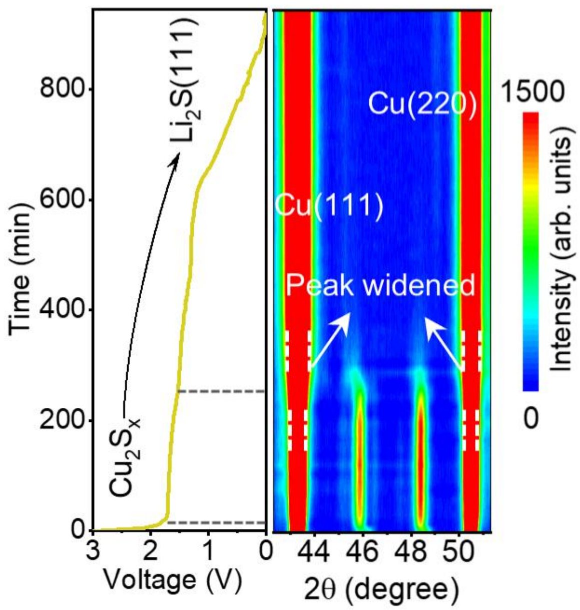

Supplementary Fig. 16| In situ XRD contour maps and corresponding discharge curve of Cu clusters formation process. The dashed lines are the positions where the peaks shift occurs, the applied current density: 0.5 mA cm^{-2}, potential cutoff: 0.01 V, temperature: 25 ± 1°C.

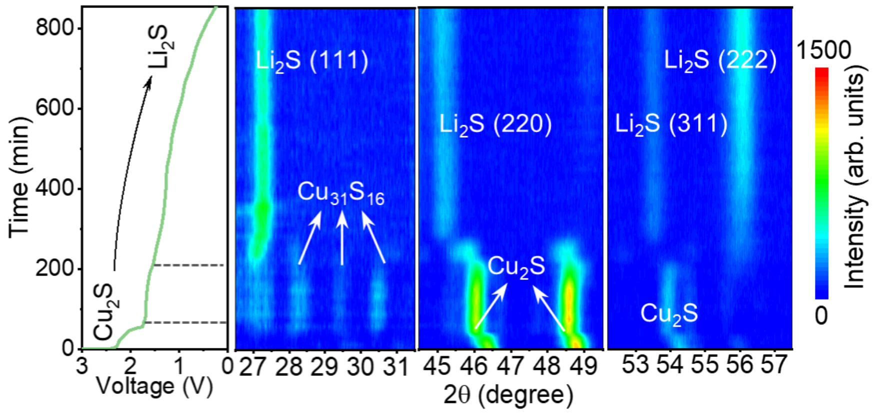

Supplementary Fig. 17| In situ XRD contour maps and corresponding discharge curve of Li_2S@Cu formation process. The dashed lines are the positions where the peaks shift occurs, the applied current density: 0.5 mA cm^{-2}, potential cutoff: 0.01 V, temperature: 25 ± 1°C.

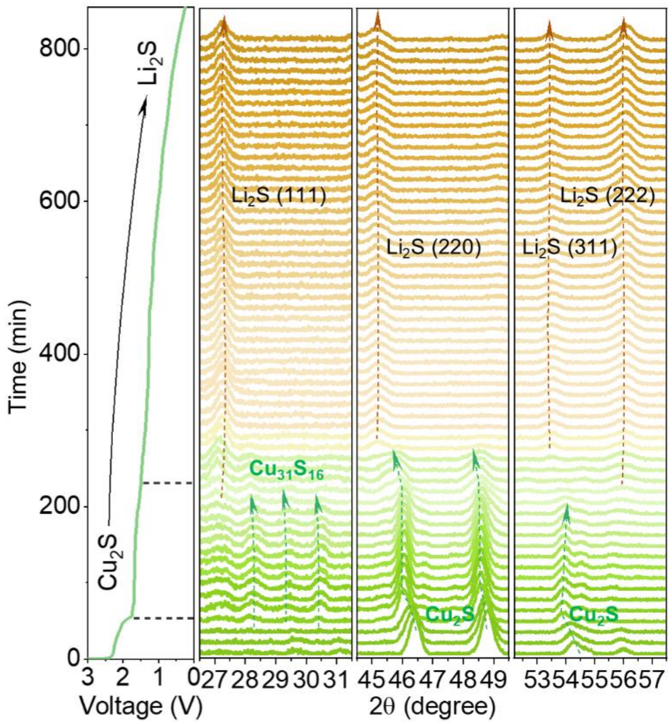

Supplementary Fig. 18| In situ XRD 1D maps and corresponding discharge curve of Li_2S@Cu formation process. The dashed lines are the positions where the peaks shift occurs, the applied current density: 0.5 mA cm^{-2}, potential cutoff: 0.01 V, temperature: 25 ± 1°C.

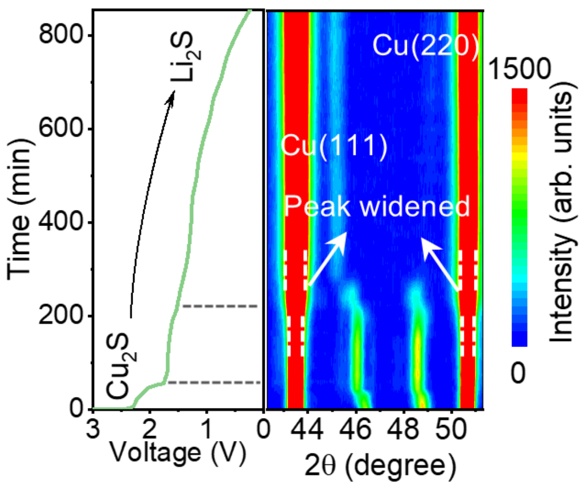

Supplementary Fig. 19| In situ XRD contour maps and corresponding discharge curve of Cu clusters formation process. The dashed lines are the positions where the peaks shift occurs, the applied current density: 0.5 mA cm^{-2}, potential cutoff: 0.01 V, temperature: 25 ± 1°C.

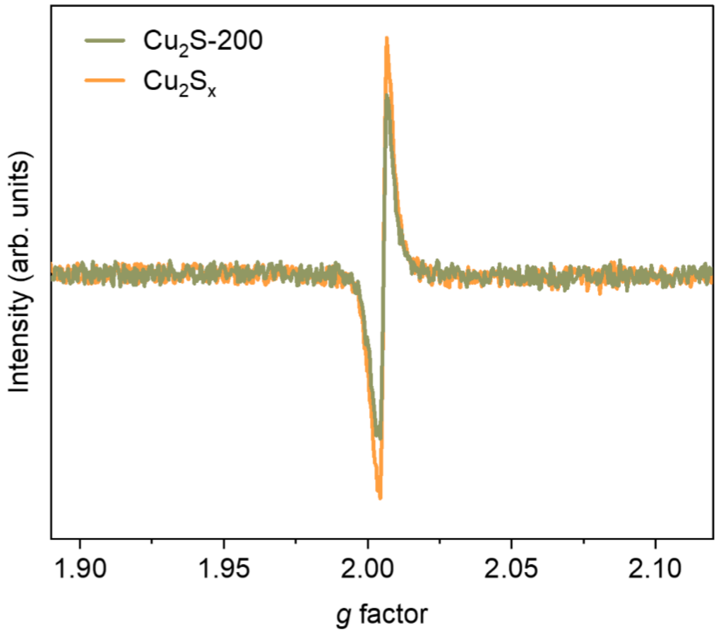

Supplementary Fig. 20| EPR spectra of Cu_2S-200 compared to Cu_2S_x.

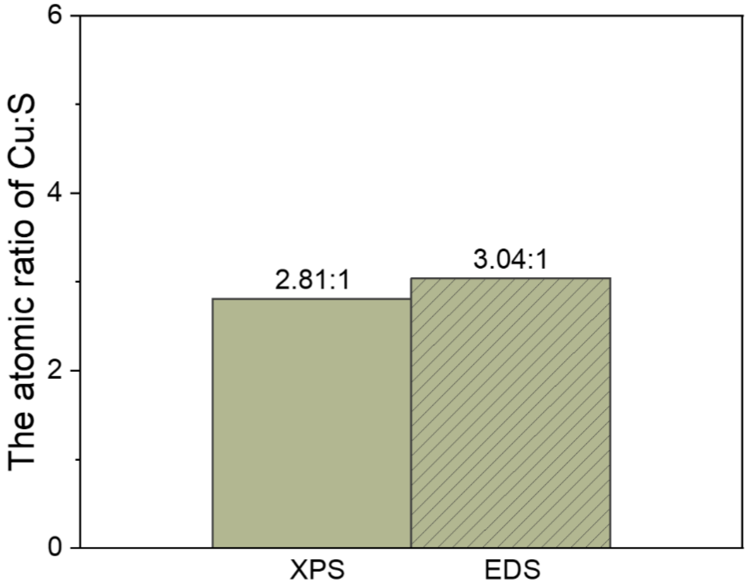

Supplementary Fig. 21| The atomic ratios of Cu:S from the XPS survey spectra and EDS data for Cu_2S-200 material.

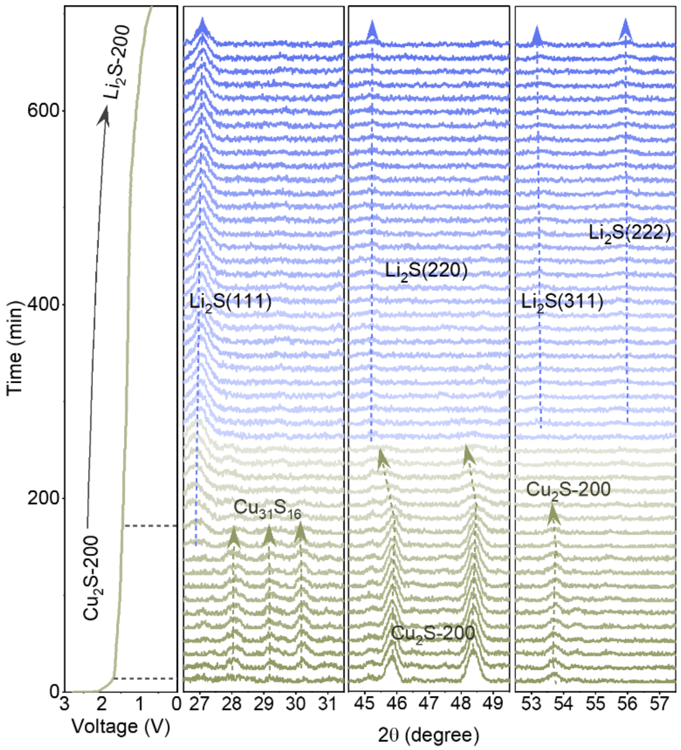

Supplementary Fig. 22| In situ XRD 1D maps and corresponding discharge curve of Li2S-200@Cu formation process (the dashed lines are the positions where the peaks shift occurs)**.**

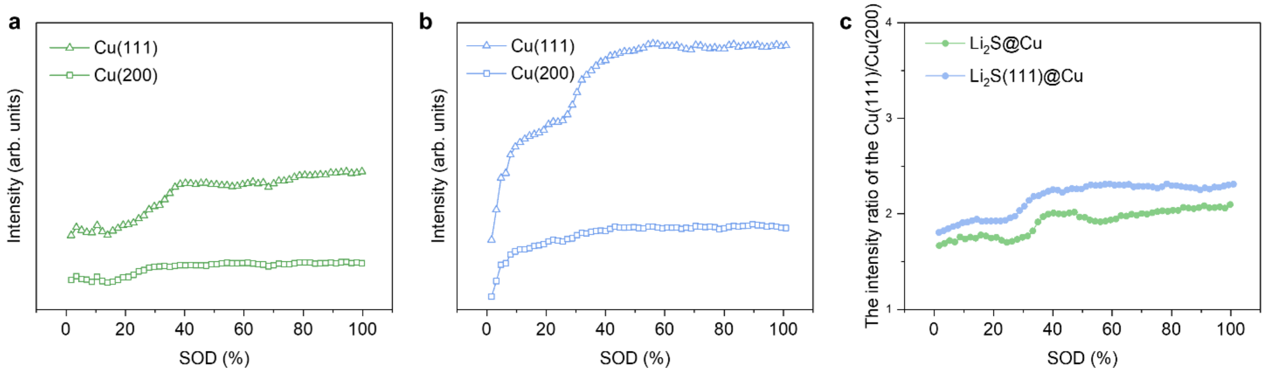

Supplementary Fig. 23| The characteristic peak intensity variations of Cu(111) and Cu(200) during the in-situ electrochemical lithiation process of Cu_2S@Cu and Cu_2S_x@Cu. (a) The peak intensity variations during the formation process of Li_2S@Cu substrate. (b) The peak intensity variations during the formation process of Li_2S(111)@Cu substrate. (c) The peak intensity ratio of the Cu(111)/Cu(200).

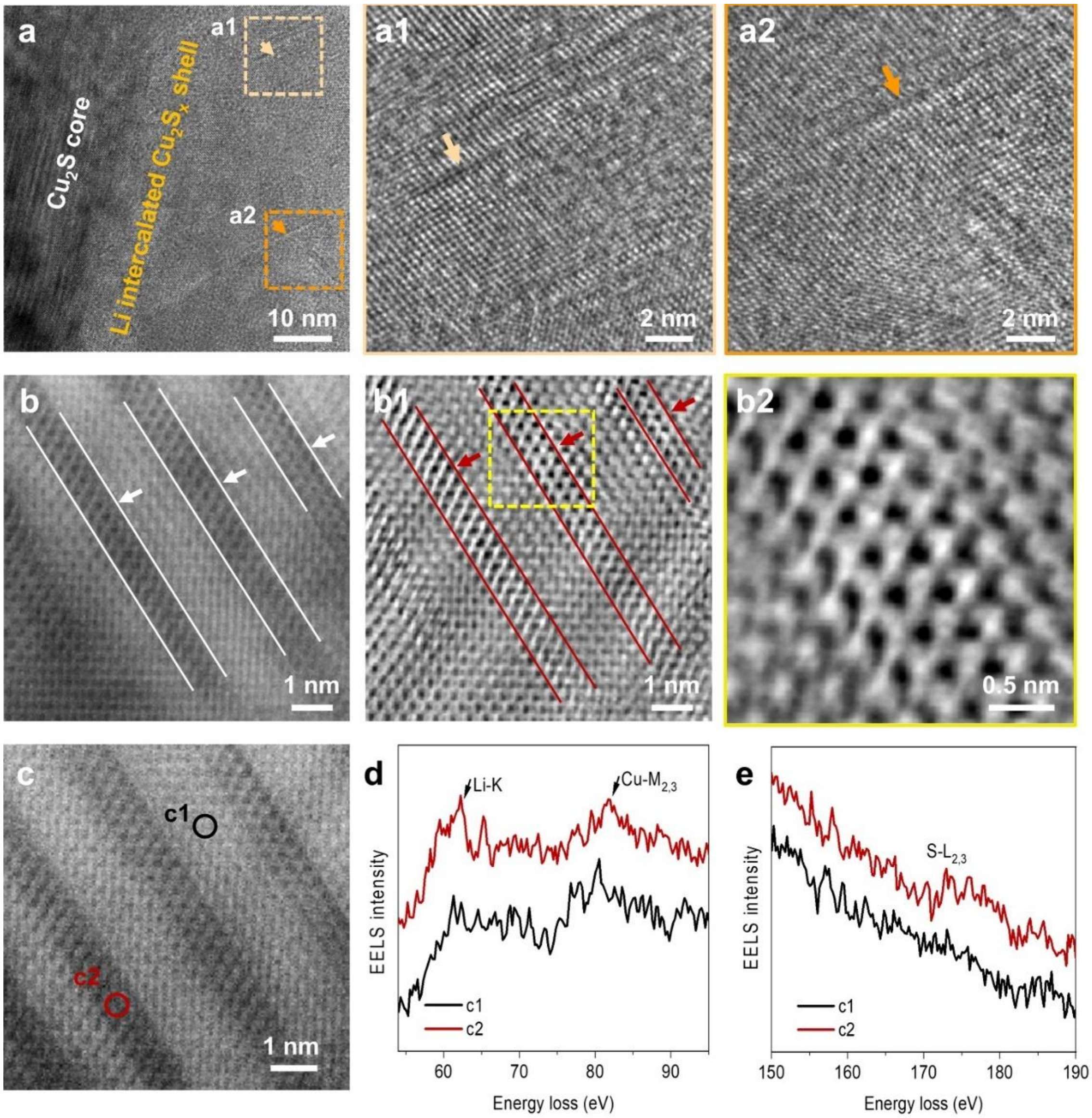

Supplementary Fig. 24| TEM characterizations of Cu_2S_x@Cu collected during lithiation. (a) HR-TEM image showing stacking faults in Li intercalated Cu_2S_x shell. The arrows in a1 and a2 region indicate the presence of a stacking fault. (b) High-resolution HAADF/iDPC-STEM images showing Cu-deficient regions in Cu_2S_x shell with Cu voids. The red solid lines and arrows highlight the Cu-deficient regions. (c) HAADF-STEM image and (d,e) STEM-EELS spectra indicating (c1) a Cu-deficient with darker contrast and (c2) S-deficient region with brighter contrast.

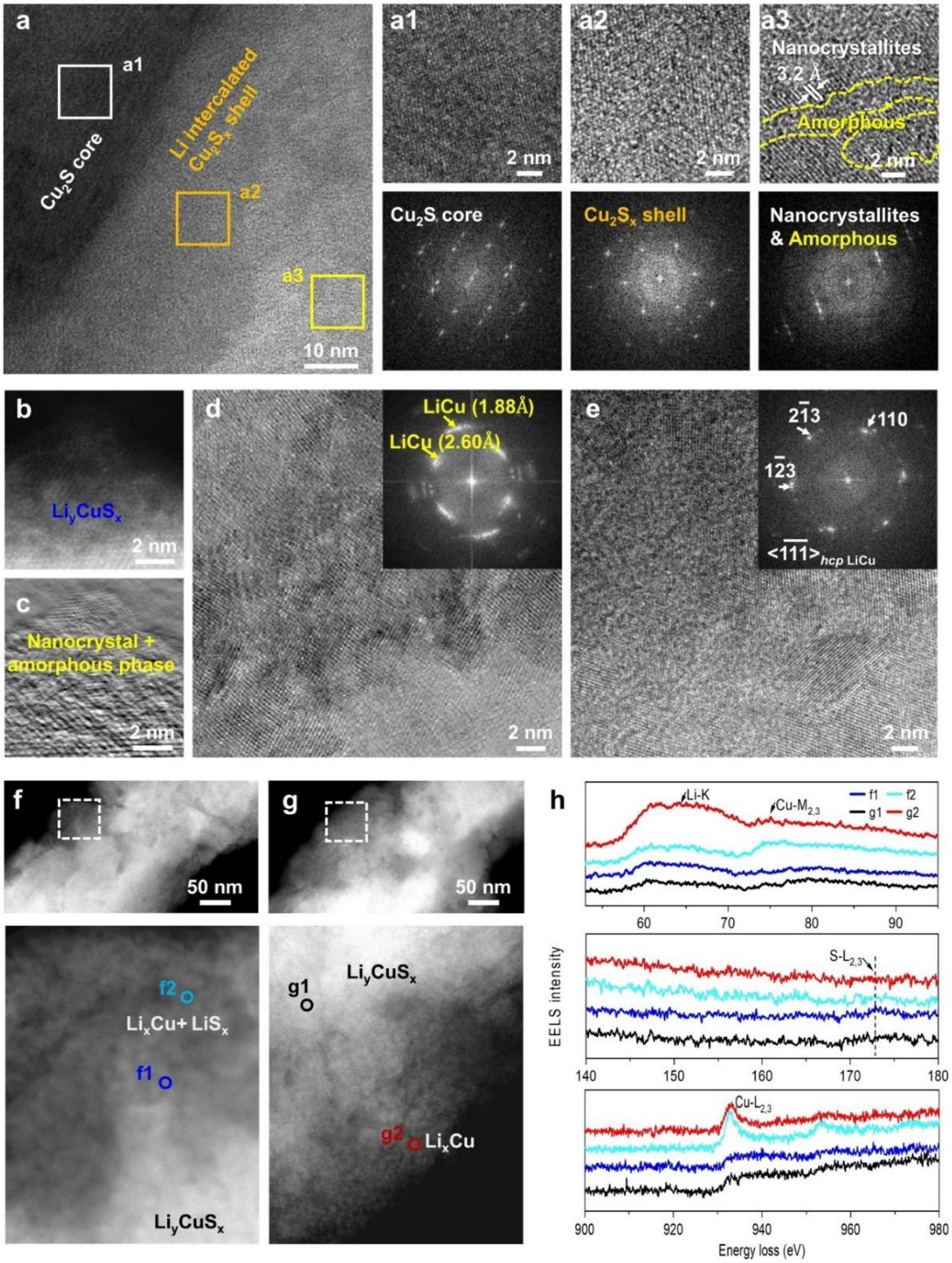

Supplementary Fig. 25| TEM characterizations of Cu_2S_x@Cu collected during lithiation. (a) TEM/HR-TEM images with the corresponding FFT diffractogram showing (a1) Cu_2S core, (a2) Li intercalated Cu_2S_x shell, (a3) nanocrystallites and amorphous phase. (b, c) HR-HAADF/iDPC-STEM image of nanocrystallites and amorphous phase on Li_yCuS_x. (d, e) HR-TEM images with the corresponding FFT diffractogram of (d) Li_xCu and (e) hcp LiCu. (f, g) HAADF-STEM images and (h) STEM-EELS spectra showing distinguished Li, Cu, and S concentrations in f1, f2, g1, and g2 region marked in f and g.

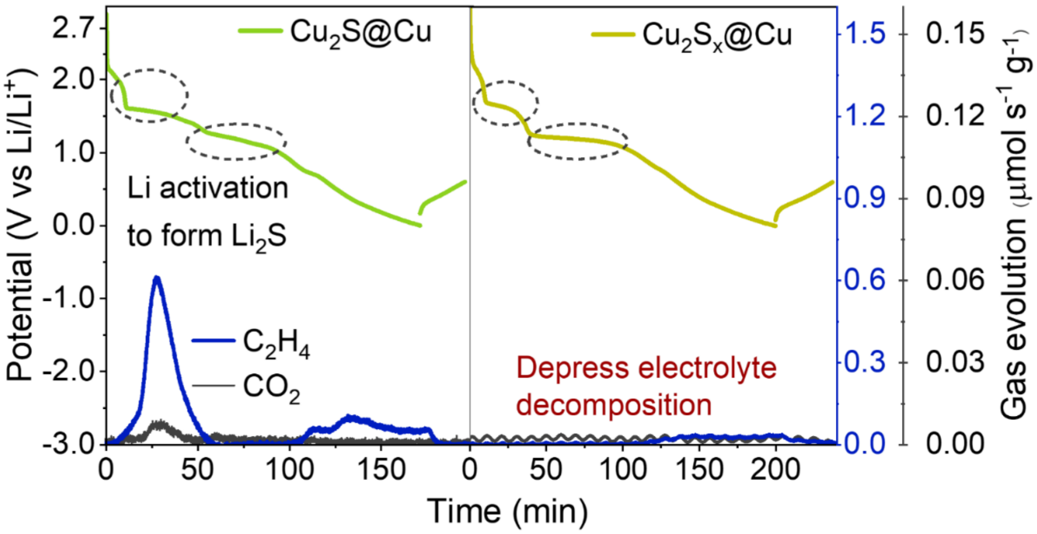

Supplementary Fig. 26| In situ OEMS tests and corresponding discharge curves of Li_2S@Cu and Li_2S(111)@Cu formation process.

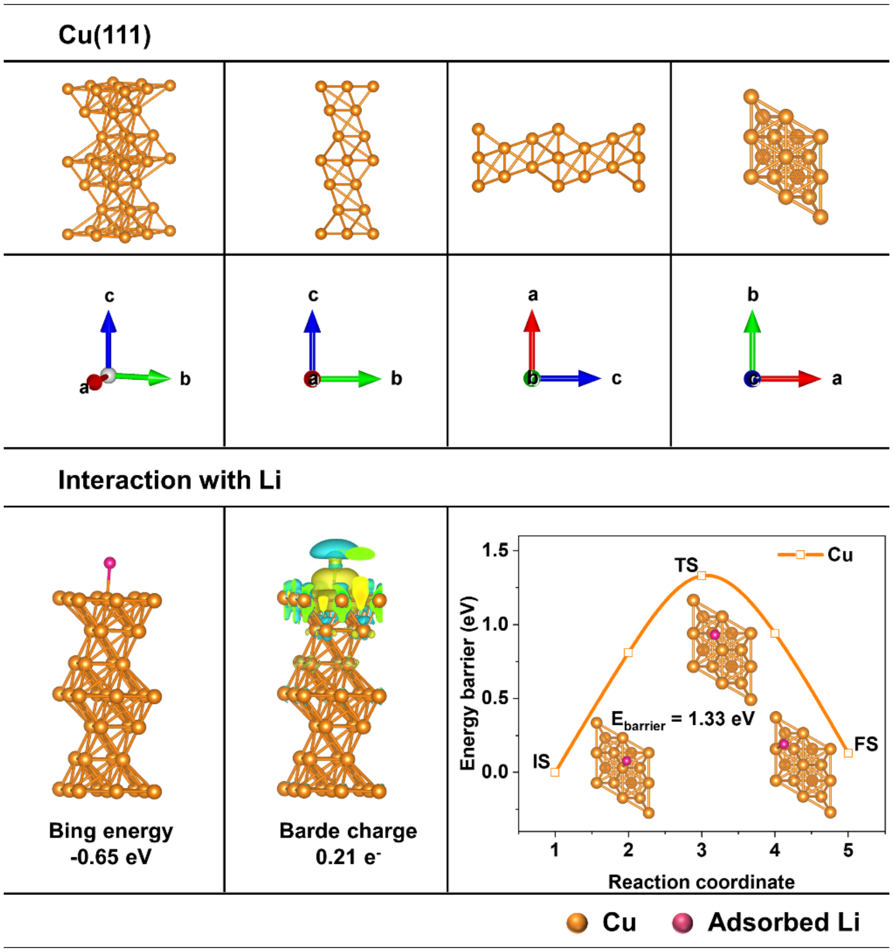

Supplementary Fig. 27| The optimized geometrical structure models of Cu(111) and the interaction with Li. Yellow: Cu; Pink: adsorbed Li.

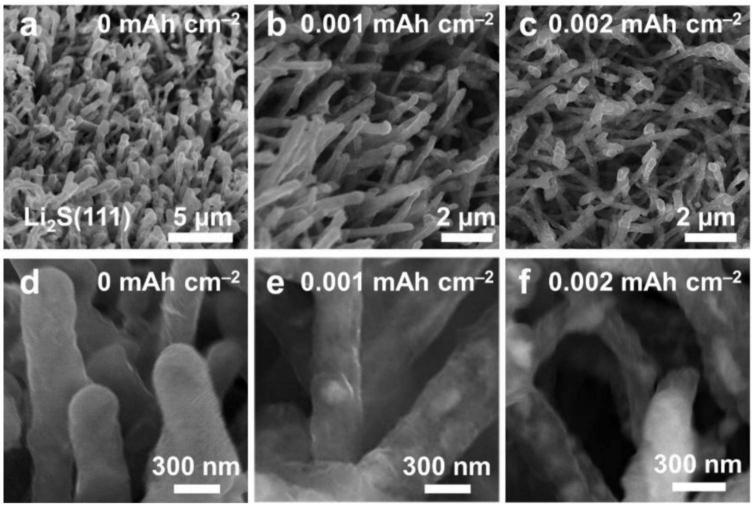

Supplementary Fig. 28| SEM images of Li deposited on Li_2S(111)@Cu substrate. (a, d) Initial Li_2S(111)@Cu substrate. (b, e) After depositing 0.001 mAh cm^{-2} and (c, f) 0.002 mAh cm^{-2} Li.

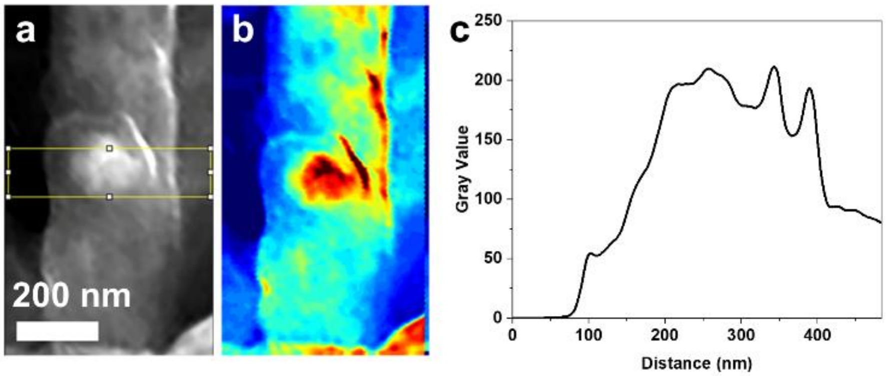

Supplementary Fig. 29| Illustration of Li particles on the Li_2S(111)@Cu substrate. (a) SEM image of 0.001 mAh cm^{-2} Li deposited on Li_2S(111)@Cu substrate (the luminous white spots). (b) The corresponding gray value intensity distribution. (c) The corresponding gray distribution of the rectangle area.

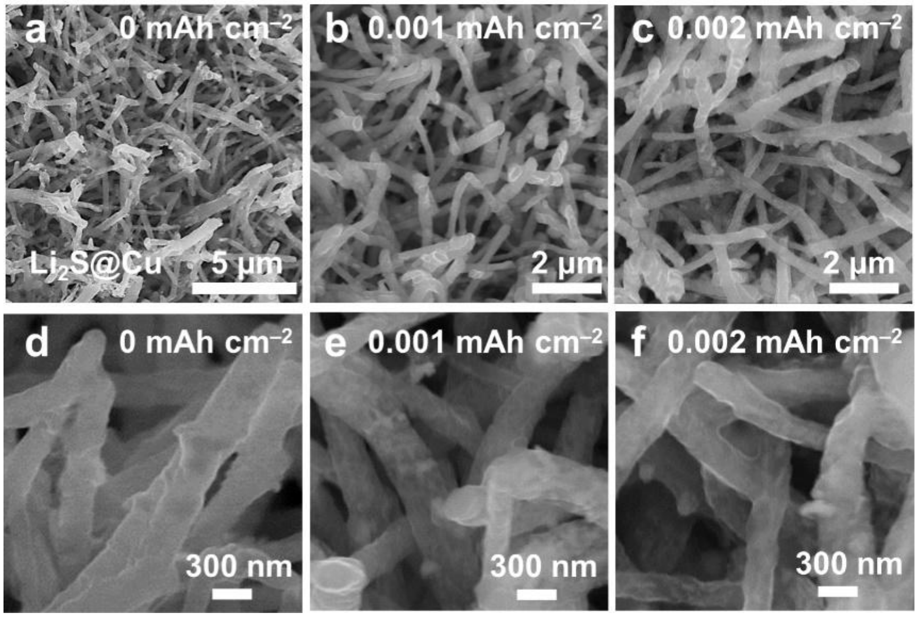

Supplementary Fig. 30| SEM images of Li deposited on Li_2S@Cu substrate. (a, d) Initial Li_2S@Cu substrate. (b, e) After depositing 0.001 mAh cm^{-2} and (c, f) 0.002 mAh cm^{-2} Li.

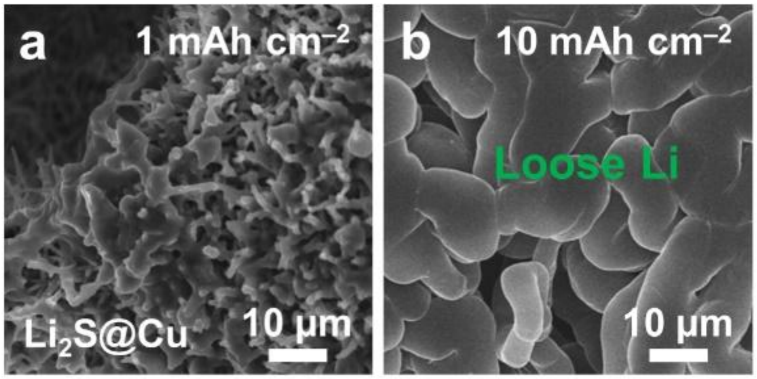

Supplementary Fig. 31| SEM images of Li deposited on Li_2S@Cu substrate with 1 and 10 mAh cm^{-2} capacities. (a) 1 mAh cm^{-2}. (b) 10 mAh cm^{-2}.

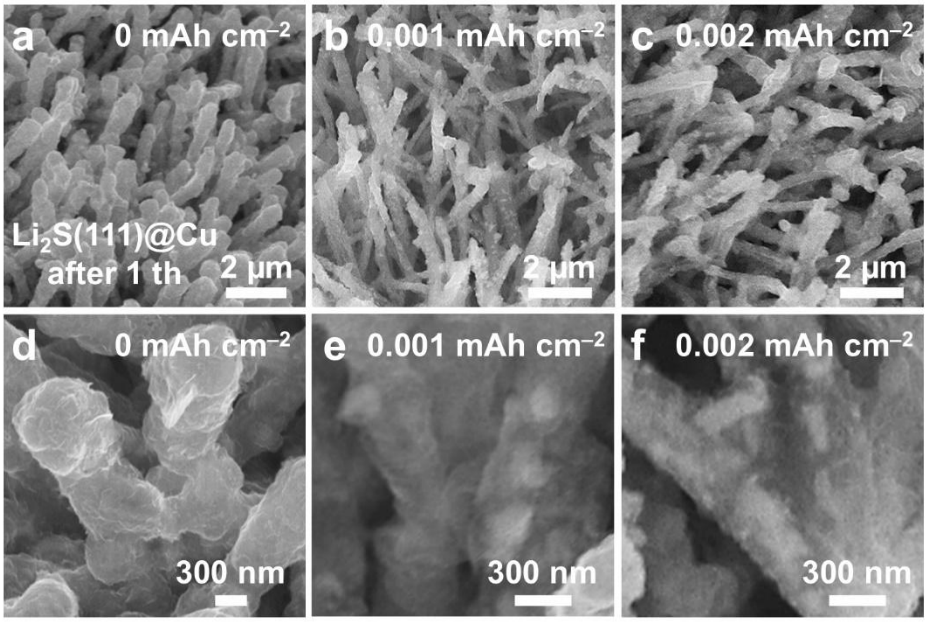

Supplementary Fig. 32| SEM images of Li deposited on Li_2S(111)@Cu substrate after 1th. (a, d) Li_2S(111)@Cu substrate after the first cycle. (b, e) After deposited 0.001 mAh cm^{-2} Li. (c, f) After deposited 0.002 mAh cm^{-2} Li.

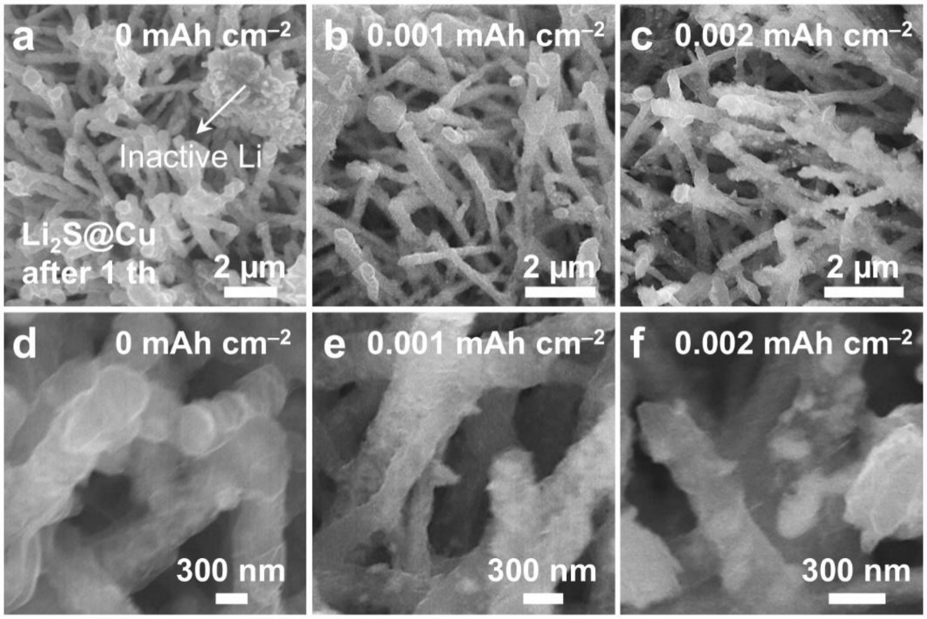

Supplementary Fig. 33| SEM images of Li deposited on Li_2S@Cu substrate after 1th. (a, d) Li_2S@Cu substrate after the first cycle. (b, e) After deposited 0.001 mAh cm^{-2} Li. (c, f) After deposited 0.002 mAh cm^{-2} Li.

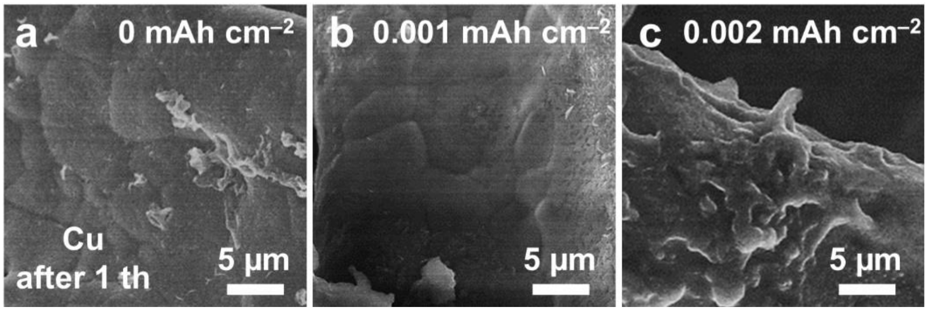

Supplementary Fig. 34| SEM images of Li deposited on Cu substrate after the first cycle. (a) Cu substrate after the first cycle. (b-c) After deposited b) 0.001 mAh cm^{-2} and c) 0.002 mAh cm^{-2} Li.

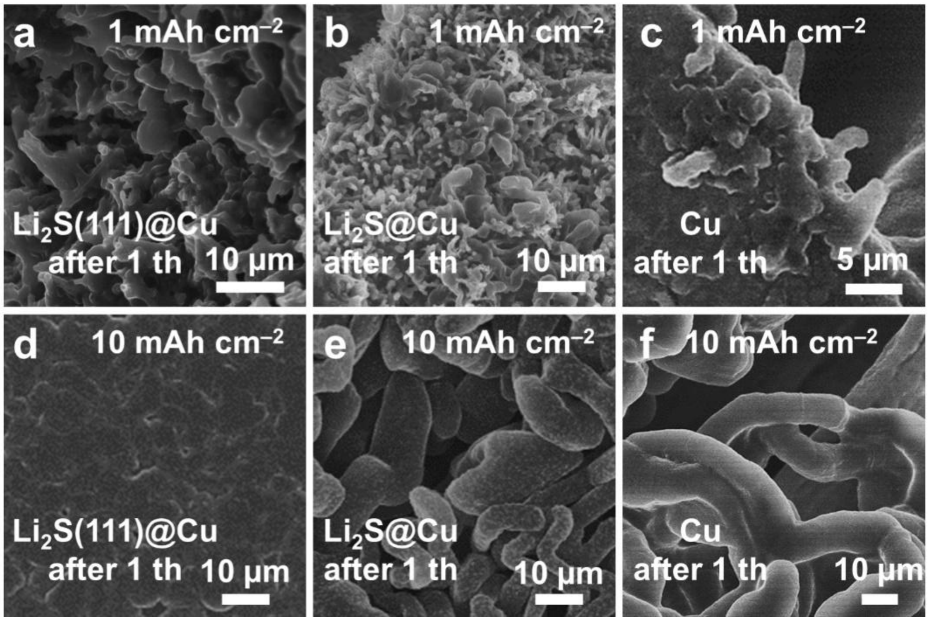

Supplementary Fig. 35| SEM images of Li deposited on different substrates after the first cycle with 1 and 10 mAh cm^{-2}. (a, d) Li_2S(111)@Cu substrate. (b, e) Li_2S@Cu substrate. (c, f) Cu substrate.

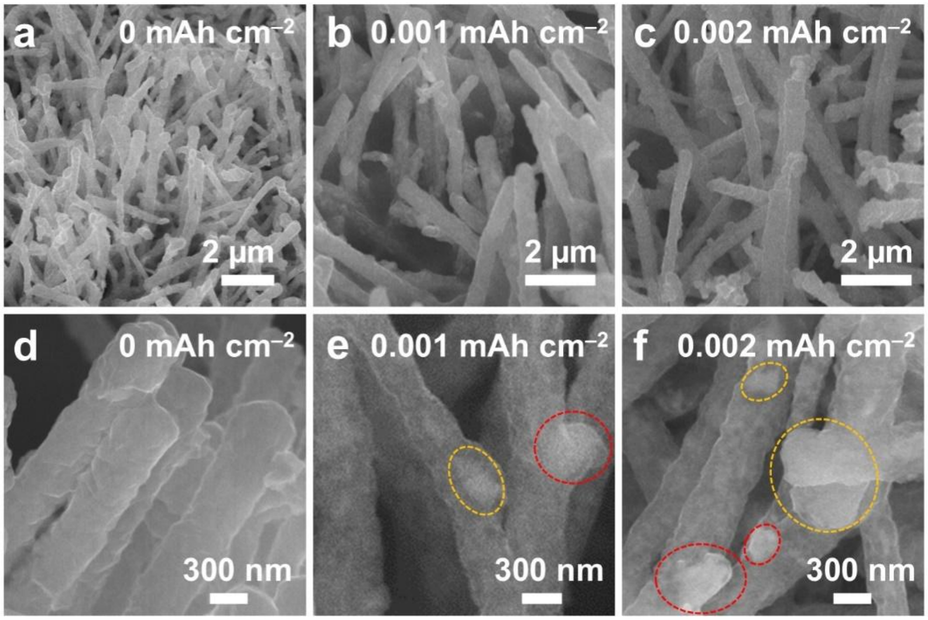

Supplementary Fig. 36| SEM images of Li deposited on Li_2S-200@Cu substrate. (a, d) Initial Li_2S-200@Cu substrate. (b, e) After depositing 0.001 mAh cm^{-2} and (c, f) 0.002 mAh cm^{-2} Li.

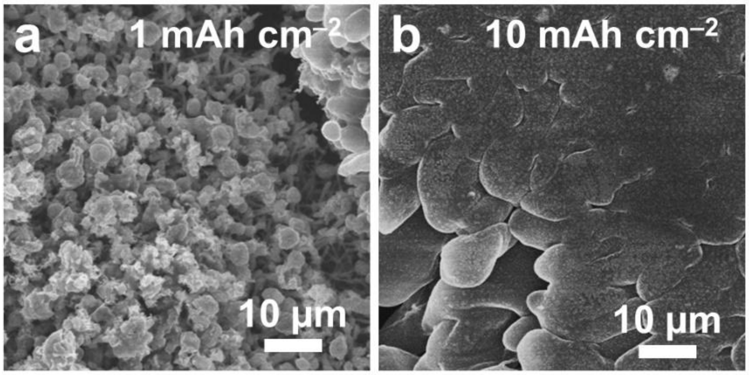

Supplementary Fig. 37| SEM images of Li deposited on Li_2S-200@Cu substrate. (a) 1 mAh cm^{-2} Li and (b) 10 mAh cm^{-2} Li.

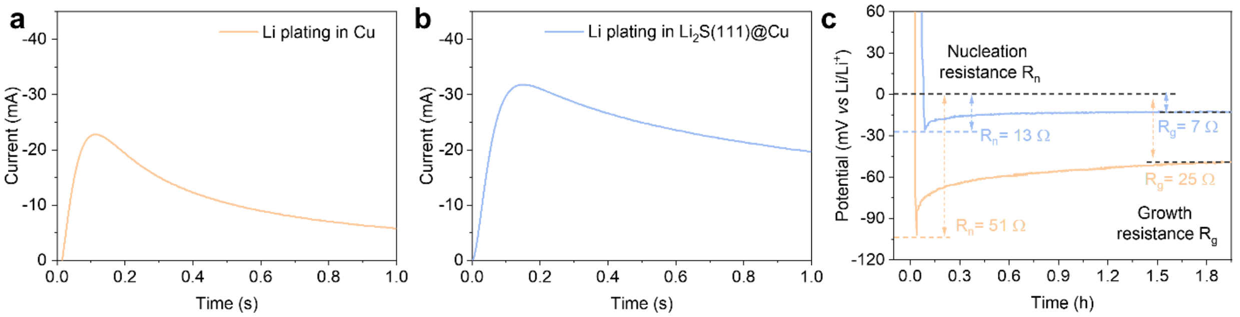

Supplementary Fig. 38| Electrochemical analysis of Li nucleation and growth on Cu and Li_2S(111)@Cu substrates. (a-b) Chronoamperometry *I*-t transients of Li deposition on a) Cu and b) Li_2S(111)@Cu substrates with an overpotential of -0.06 V. (c) Potential as a function of time curves during the initial Li plating at 1 mA cm^{-2}.

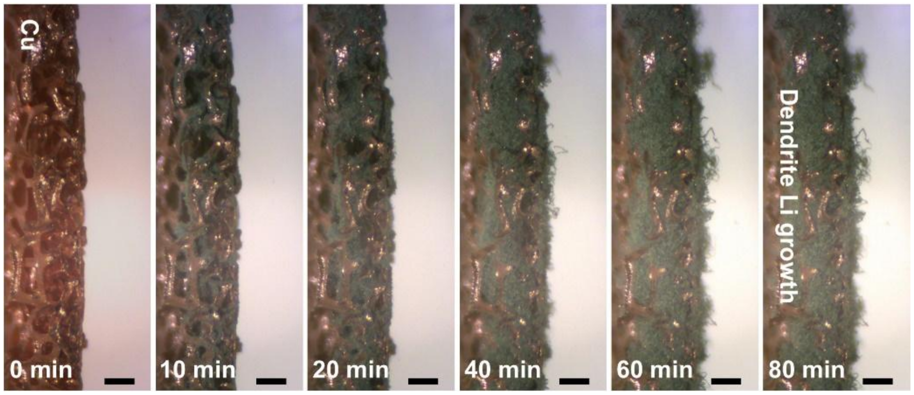

Supplementary Fig. 39| In situ optical microscopy images of Li deposited on Cu substrate. Current density: 3.5 mAh cm^{-2}, the scale bars of the images are 2 μm.

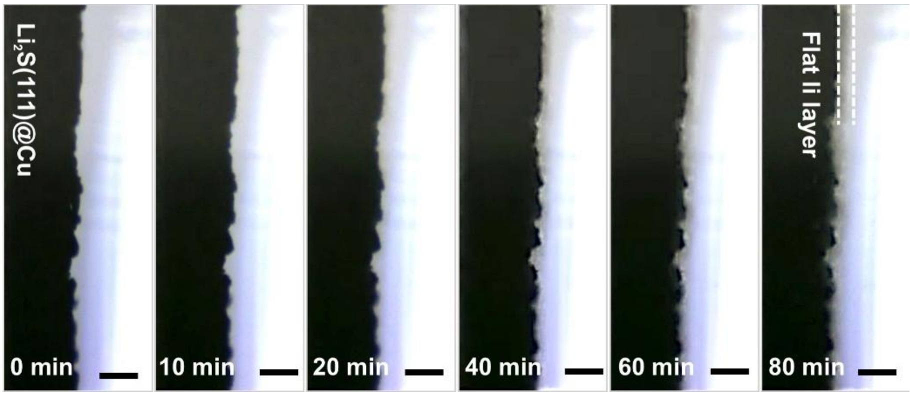

Supplementary Fig. 40| In situ optical microscopy images of Li deposited on $Li_2S(111)$@Cu substrate. Current density: 3.5 mAh cm^{-2}, the scale bars of the images are 2 μm.

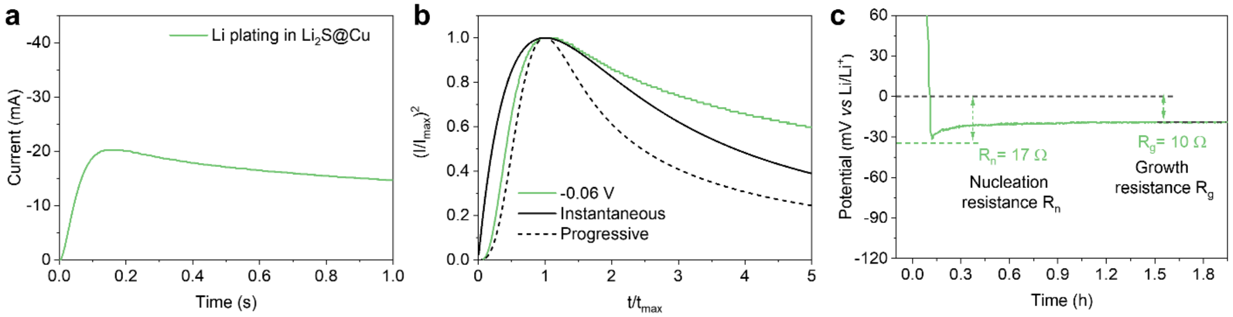

Supplementary Fig. 41| Electrochemical analysis of Li nucleation and growth on Li_2S@Cu substrate. (a) Chronoamperometry *I*-t transient of Li deposition on Li_2S@Cu substrate with an overpotential of -0.06 V. (b) The corresponding dimensionless plots of *I*-t transient. (c) Potential curve during the initial Li plating at 1 mA cm^{-2}.

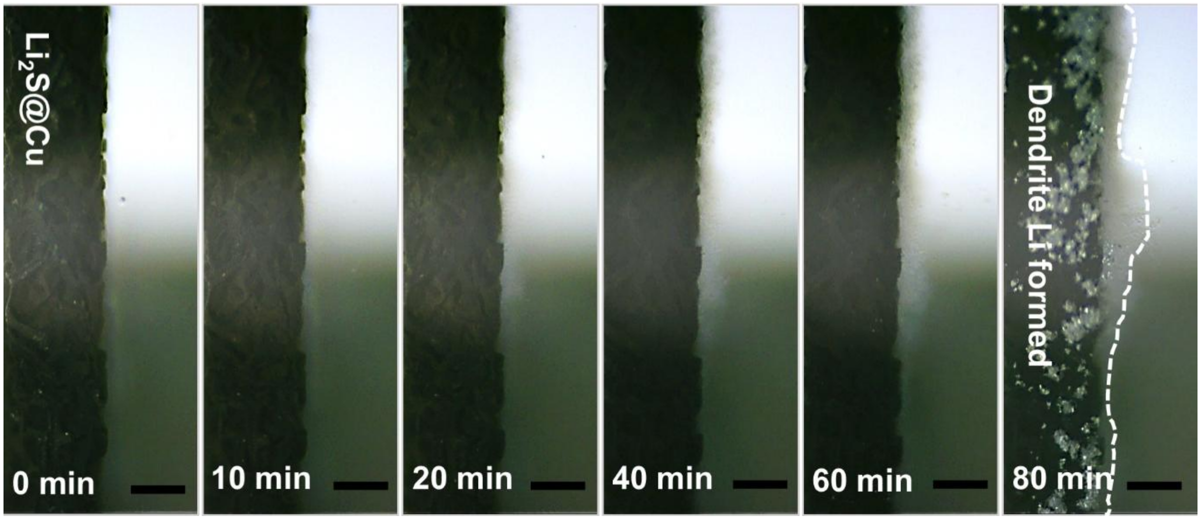

Supplementary Fig. 42| In situ optical microscopy images of Li deposited on Li_2S@Cu substrate. Current density: 3.5 mAh cm^{-2}, the scale bars of the images are 2 μm.

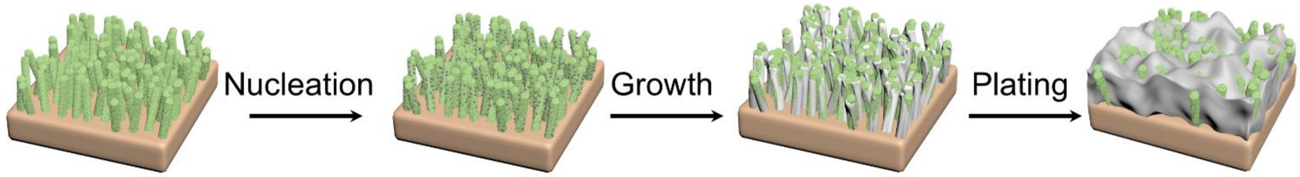

Supplementary Fig. 43| A schematic illustration depicting the loose growth of Li deposited on Li_2S@Cu substrate.

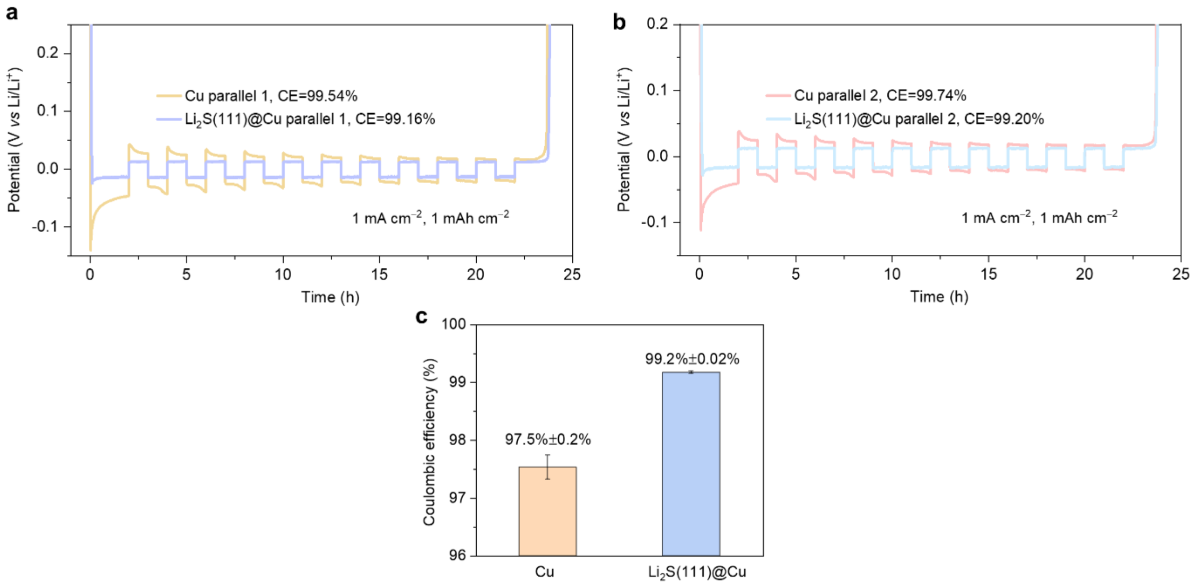

Supplementary Fig. 44| CE of Li||substrate half-cells with Cu and Li_2S(111)@Cu substrates. (a) Potential profiles of CE test under a partial-stripping mode at 1 mA cm^{-2} with 1 mAh cm^{-2} of parallel experiment 1. (b) Potential profiles of CE test under a partial-stripping mode at 1 mA cm^{-2} with 1 mAh cm^{-2} of parallel experiment 2. (c) CE values. Error bars represent s.d.

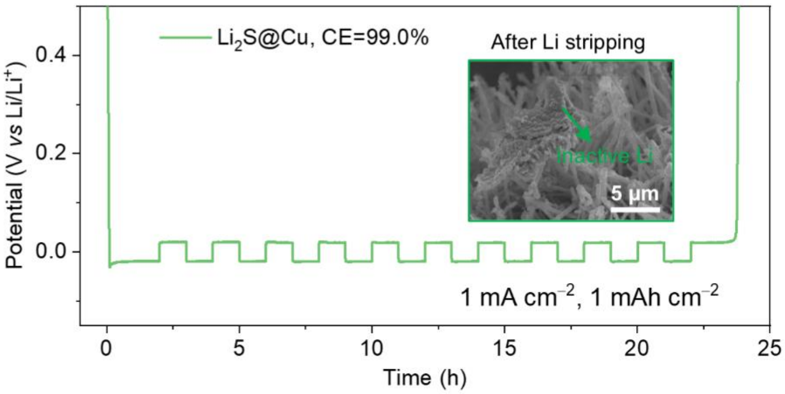

Supplementary Fig. 45| Coulombic efficiency (CE) test of half cells utilizing Li_2S@Cu substrate, with the inset showing a SEM image of the substrate after complete Li stripping.

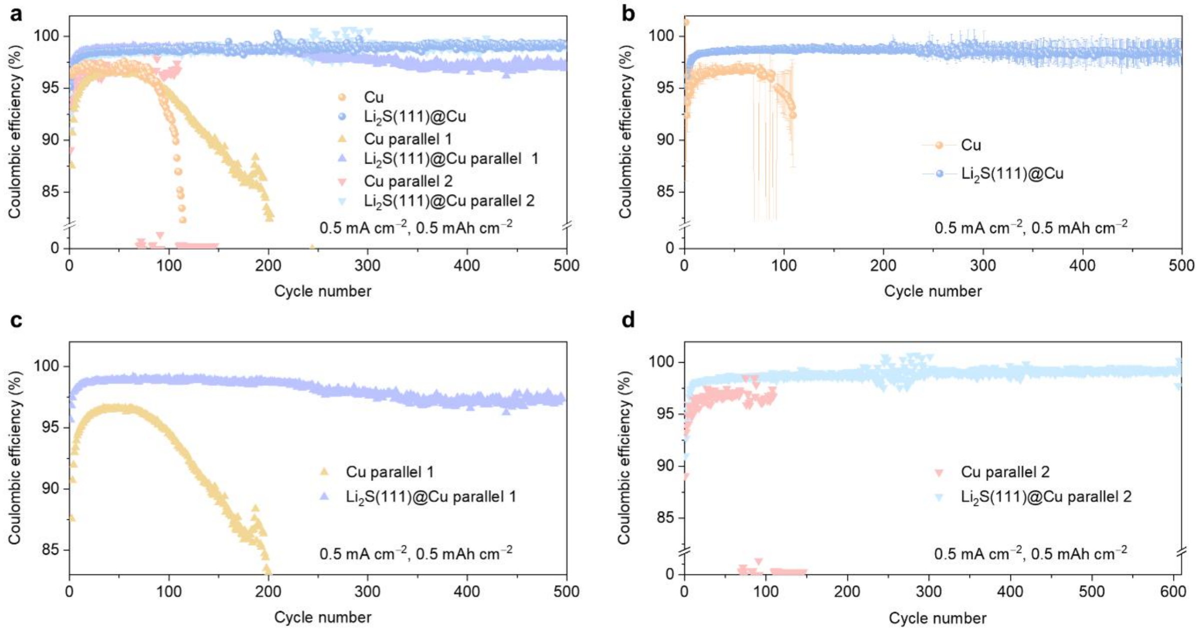

Supplementary Fig. 46| Parallel experiments of CE test under a plating-full-stripping mode at 0.5 mA cm^{-2} with 0.5 mAh cm^{-2}. (a) CEs versus cycle number. (b) CE versus cycle number in manuscript. (c) CE versus cycle number of parallel experiment 1. (d) CE versus cycle number of parallel experiment 2.

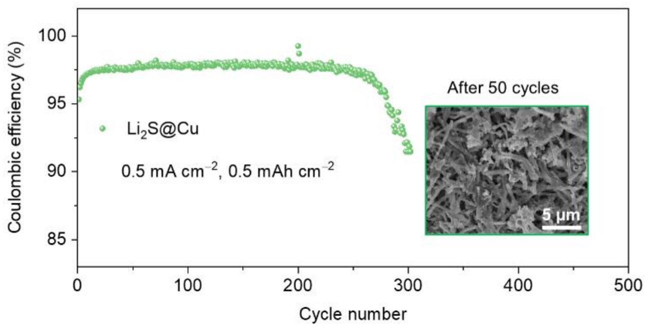

Supplementary Fig. 47| CE test of the Li_2S@Cu substrate cell was conducted in a plating-full-stripping mode, and the inset features a SEM image of the substrate structure after 50 cycles.

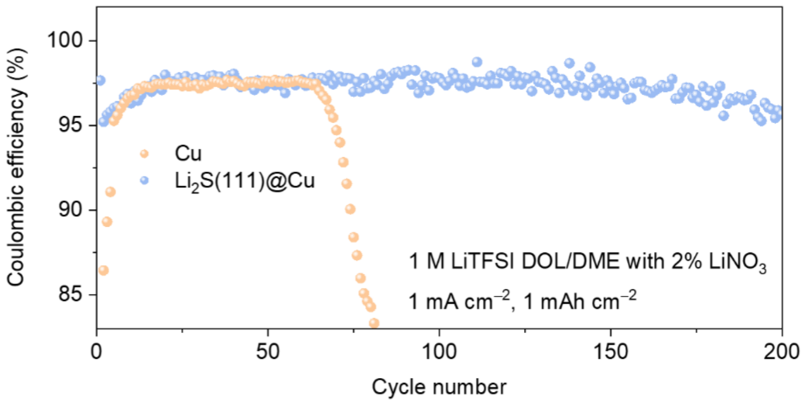

Supplementary Fig. 48| CE versus cycle number of Cu and Li_2S(111)@Cu substrate cells at 1 mA cm^{-2} and 1 mAh cm^{-2}.

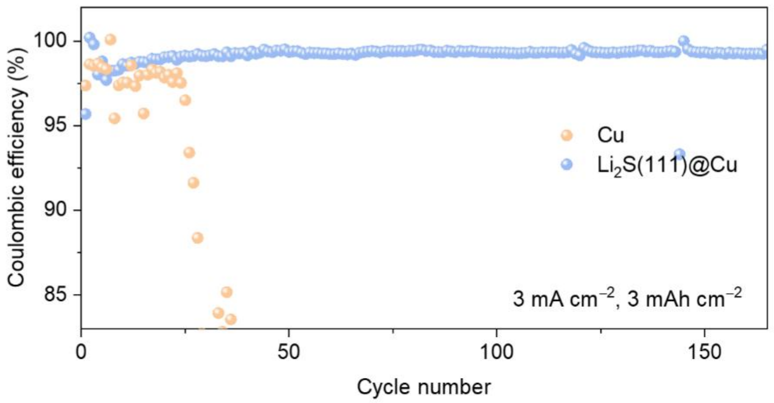

Supplementary Fig. 49| CE versus cycle number of Cu and Li_2S(111)@Cu substrate cells at 3 mA cm^{-2} and 3 mAh cm^{-2}.

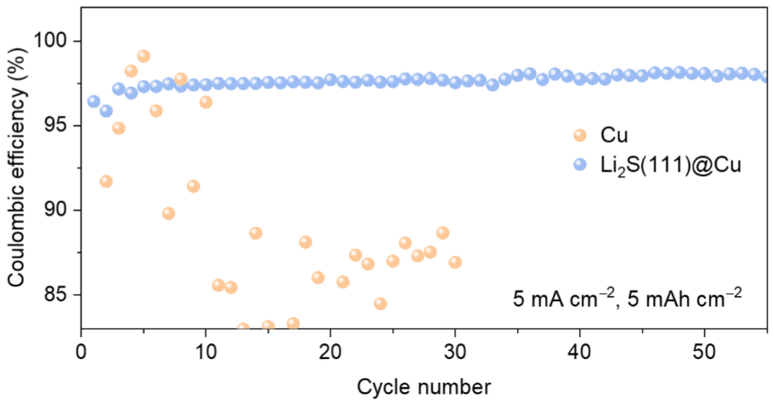

Supplementary Fig. 50| CE versus cycle number of Cu and $Li_2S(111)$@Cu substrate cells at 5 mA cm^{-2} and 5 mAh cm^{-2}.

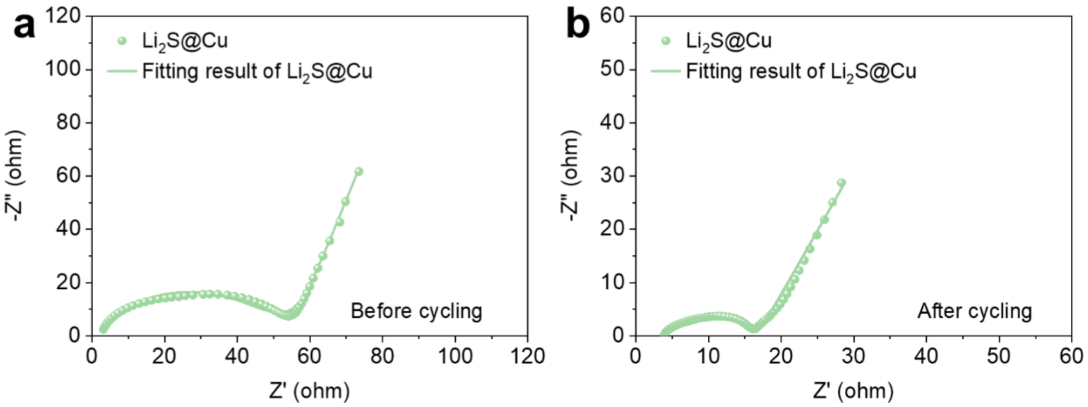

Supplementary Fig. 51| Nyquist plots of Li||substrate half cells assembled with Li_2S@Cu substrate before and after CEs tests in Supplementary Fig. 34. (a) Before CE test. (b) After CE test.

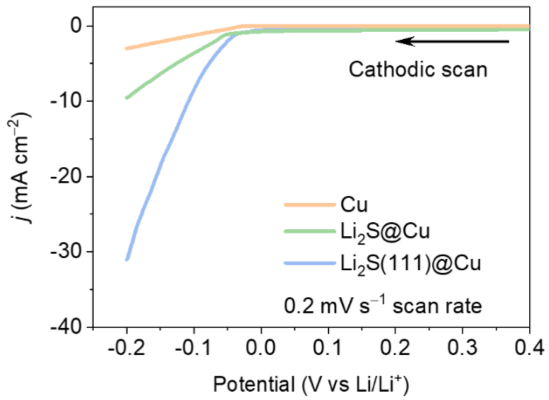

Supplementary Fig. 52| LSV curves obtained from Li||substrate half cells. Scan rate of 0.2 mV s^{-1}.

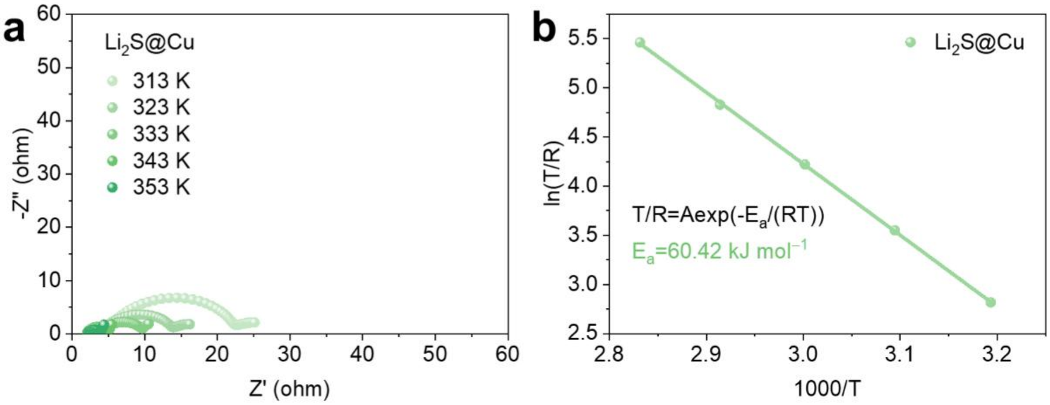

Supplementary Fig. 53| The activation energy analysis for Li deposition on Li_2S@Cu substrate. (a) EIS spectra of Li||Li_2S@Cu half cells obtained at different temperatures. (b) The corresponding Arrhenius plots behavior of the resistance.

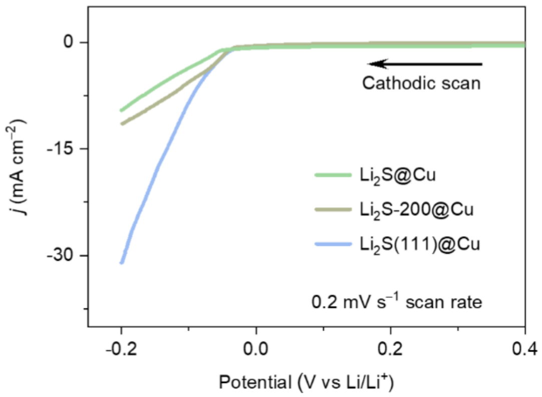

Supplementary Fig. 54| LSV curves of the half-cell based on Li_2S-200@Cu substrate, compared to those with Li_2S@Cu and $Li_2S(111)$@Cu substrates.

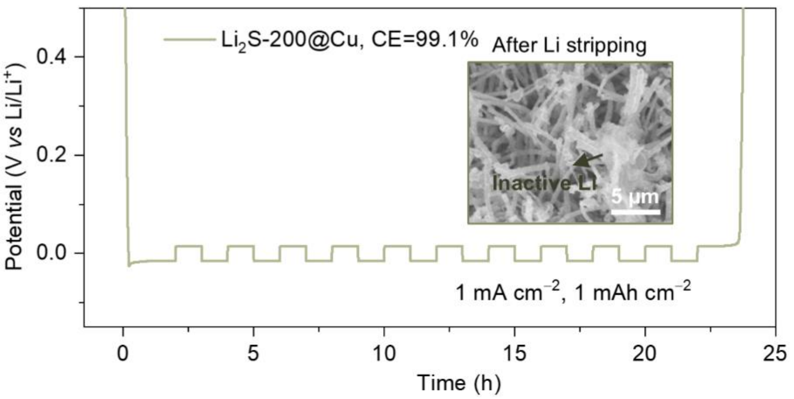

Supplementary Fig. 55| CE test of half cells utilizing Li_2S-200@Cu substrate, with the inset showing a SEM image of the substrate after complete Li stripping.

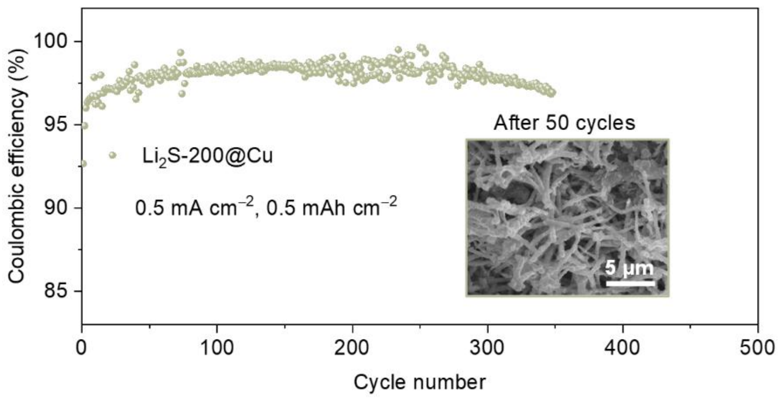

Supplementary Fig. 56| CE test of the Li_2S-200@Cu substrate cell was conducted in a plating-full-stripping mode, and the inset features a SEM image of the substrate structure after 50 cycles.

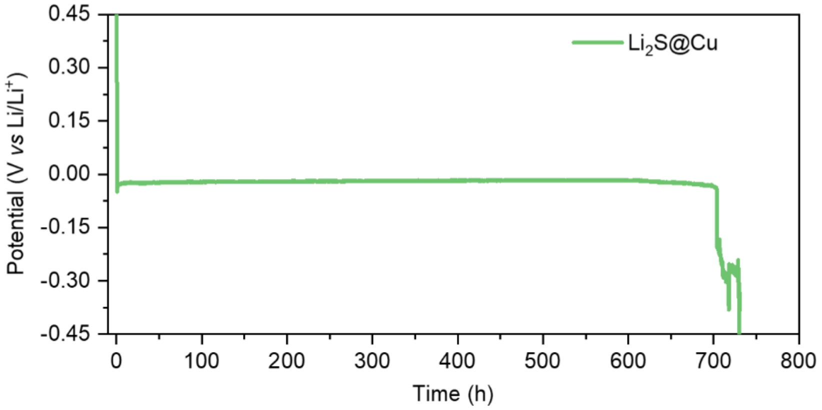

Supplementary Fig. 57| Potential-time curves of short-circuit test for Li||Li_2S@Cu cell. Current density: 0.5 mA cm^{-2}.

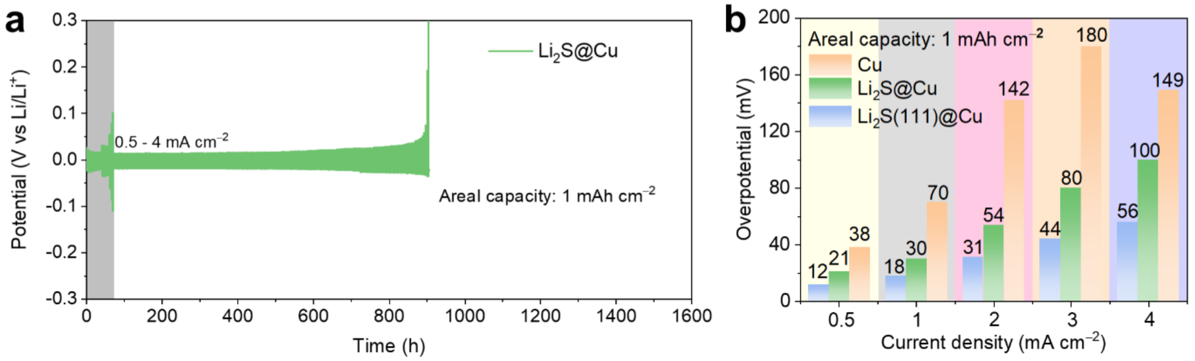

Supplementary Fig. 58| Rate performance of the symmetric cell based on different substrates. (a) The rate performance of the symmetric cell based on Li_2S@Cu substrate at different current densities of 0.5, 1, 2, 3 and 4 mA cm^{-2} with a fixed capacity of 1 mAh cm^{-2}. (b) The "overpotential" histograms of symmetric cells based on Cu, Li_2S@Cu, and Li_2S(111)@Cu substrates at different current densities ranging from 0.5 to 4 mA cm^{-2}.

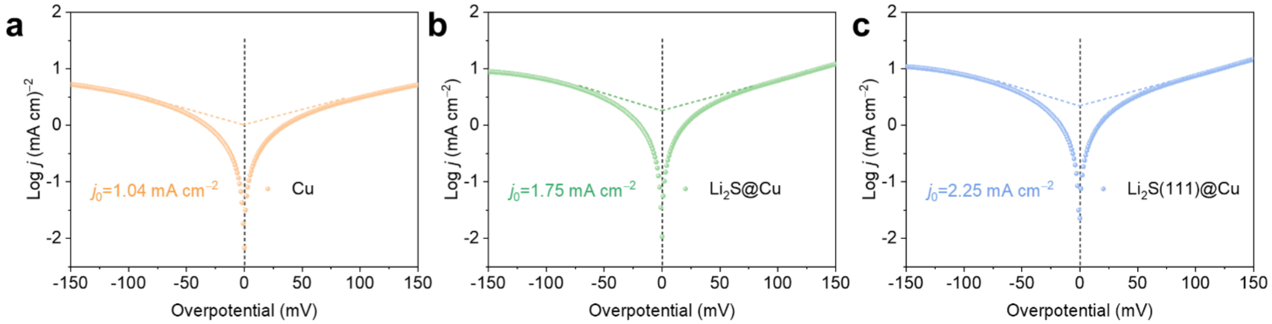

Supplementary Fig. 59| Tafel plots of symmetric cells based on different substrates. (a) Cu. (b) Li_2S@Cu and (c) Li_2S(111)@Cu substrates.

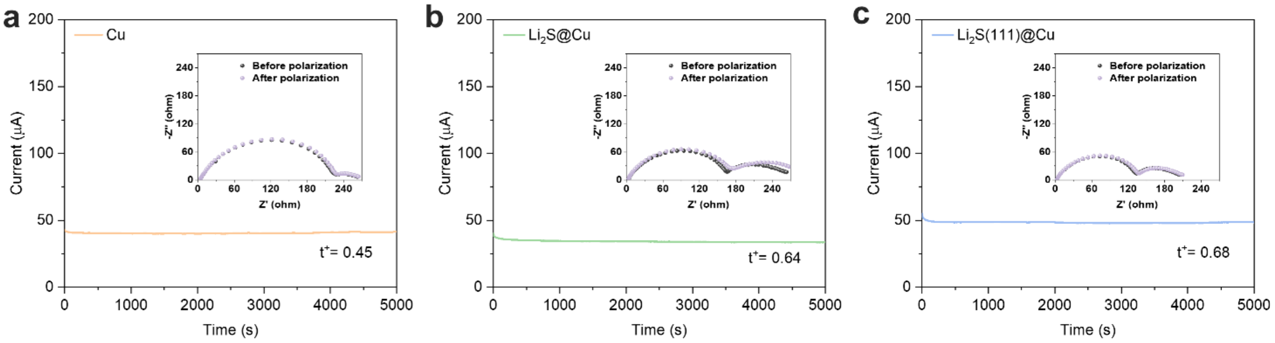

Supplementary Fig. 60| Steady-state current under 10 mV polarization for different substrates, inset shows EIS measurements before and after polarization. (a) Cu. (b) Li_2S@Cu. (c) Li_2S(111)@Cu.

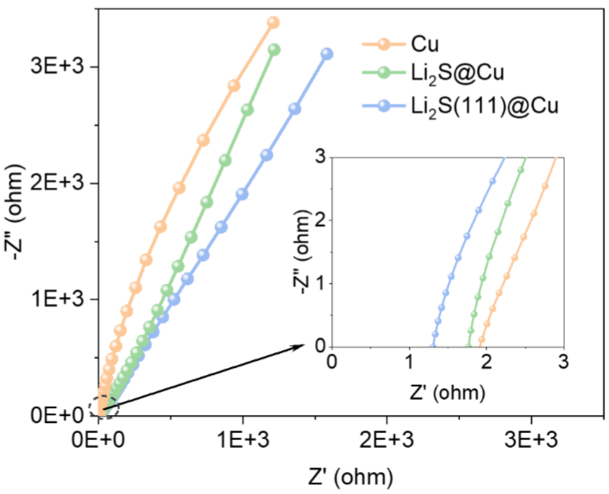

Supplementary Fig. 61| Nyquist plots of different substrates sandwiched by two stainless plates of steel.

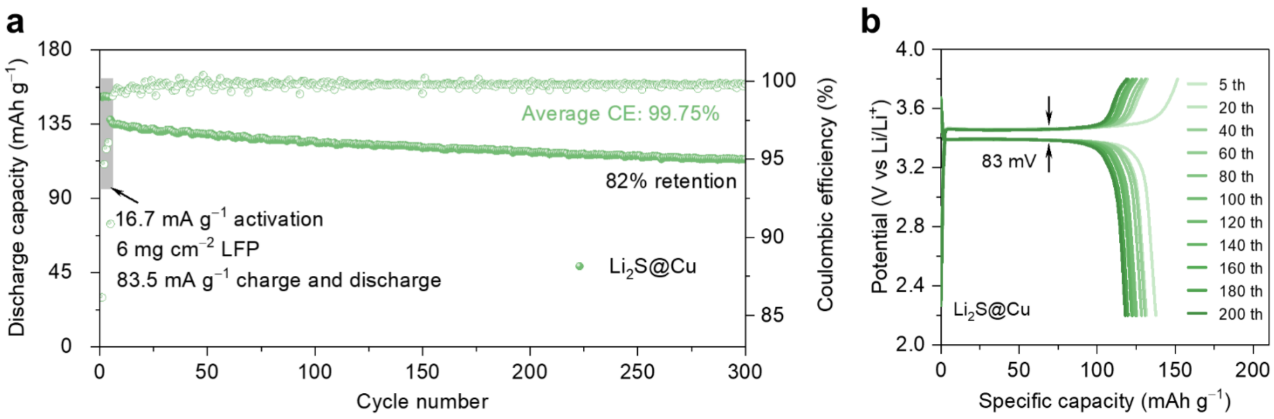

Supplementary Fig. 62| The electrochemical performance of LFP full cell based on Li_2S@Cu substrate. (a) Discharge capacity retention of Li-substrate||LFP full cells at 83.5 mA g^{-1} (1 C = 167 mA g^{-1}). (b) Corresponding galvanostatic charge/discharge profiles of LFP full cells at different cycles.

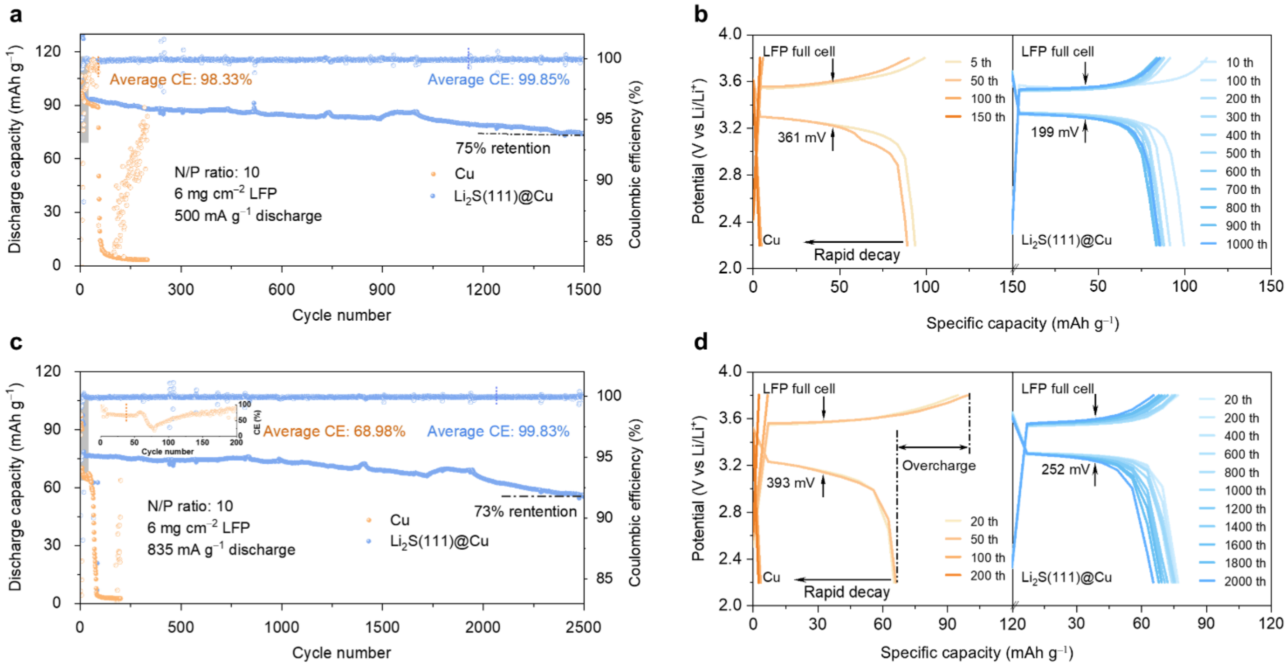

Supplementary Fig. 63| Electrochemical performance of LFP full cells based on Cu and Li_2S(111)@Cu substrates at high rates. (a) Discharge capacity retention of Li-substrate||LFP full cells at 500 mA g^{-1}. (b) Corresponding GDC profiles of LFP full cells in (a) at various cycles. (c) Discharge capacity retention of Li-substrate||LFP full cells at 835 mA g^{-1}. (d) Corresponding GDC profiles of LFP full cells in (c) at various cycles. 1 C = 167 mA g^{-1}.

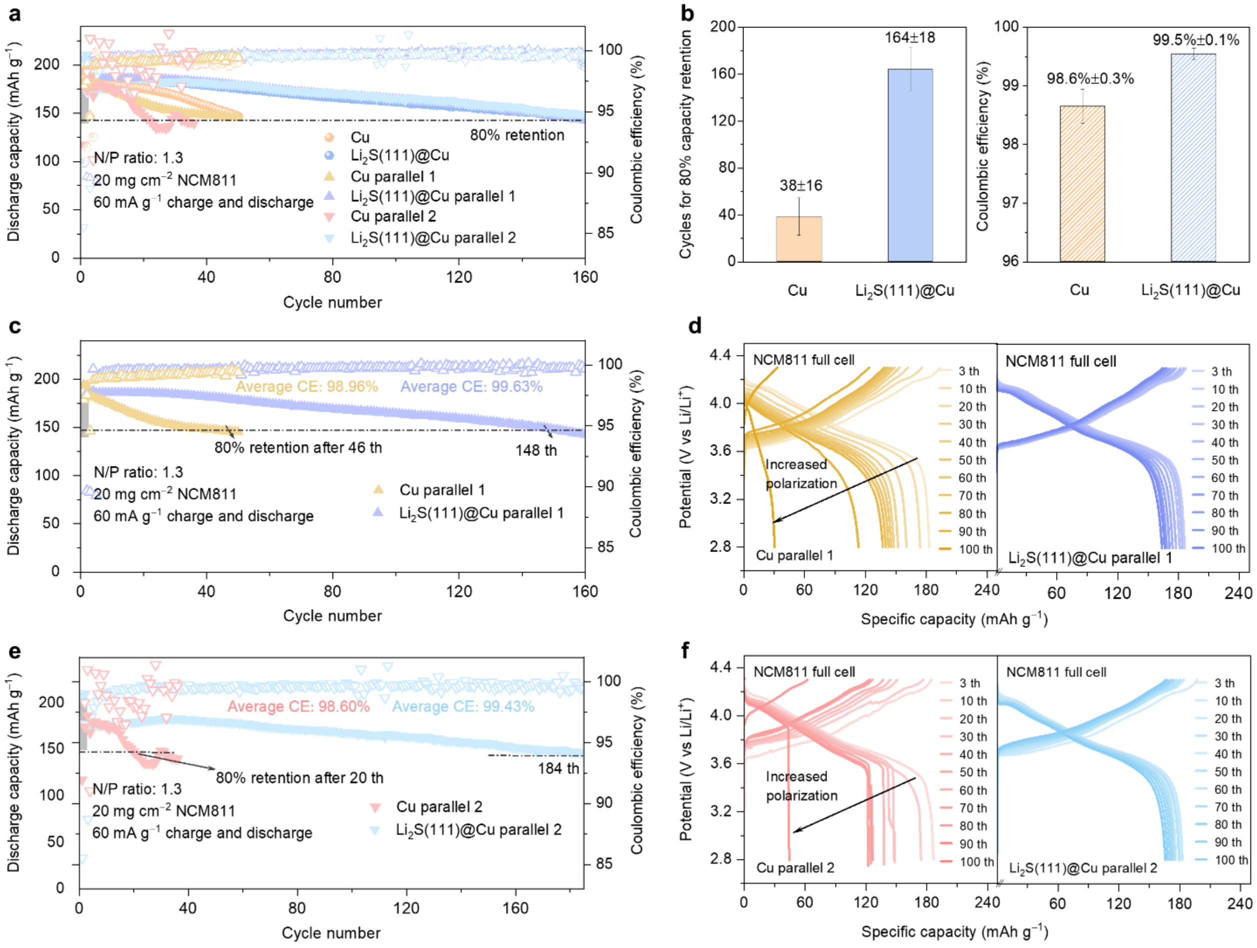

Supplementary Fig. 64| Parallel experiments of NCM811 full cells based on Cu and Li_2S(111)@Cu substrates. (a) Discharge capacity retention at 60 mA g^{-1}. (b) Cycle number for 80% capacity retention and average CE values. Error bars represent s.d. (c) Discharge capacity retention at 60 mA g^{-1} of parallel experiment 1. (d) Corresponding GDC profiles of parallel full cell 1 at different cycles. (e) Discharge capacity retention at 60 mA g^{-1} of parallel experiment 2. (f) Corresponding GDC profiles of parallel full cell 2 at different cycles. 1 C = 200 mA g^{-1}.

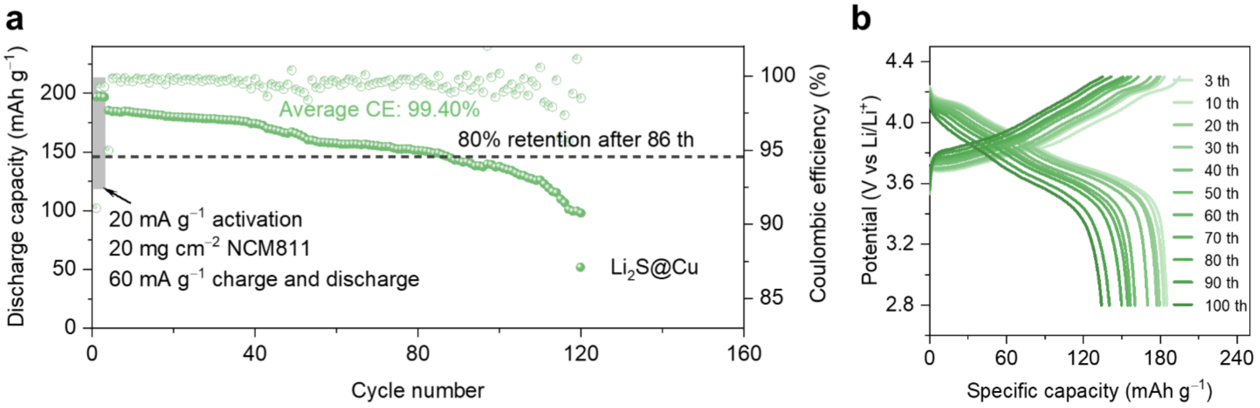

Supplementary Fig. 65| The electrochemical performance of the NCM811 full cell based on Li_2S@Cu substrate. (a) Discharge capacity retention of Li-substrate||NCM811 full cells at 60 mA g^{-1} (1 C = 200 mA g^{-1}). (b) Corresponding galvanostatic charge/discharge profiles of NCM811 full cells at different cycles.

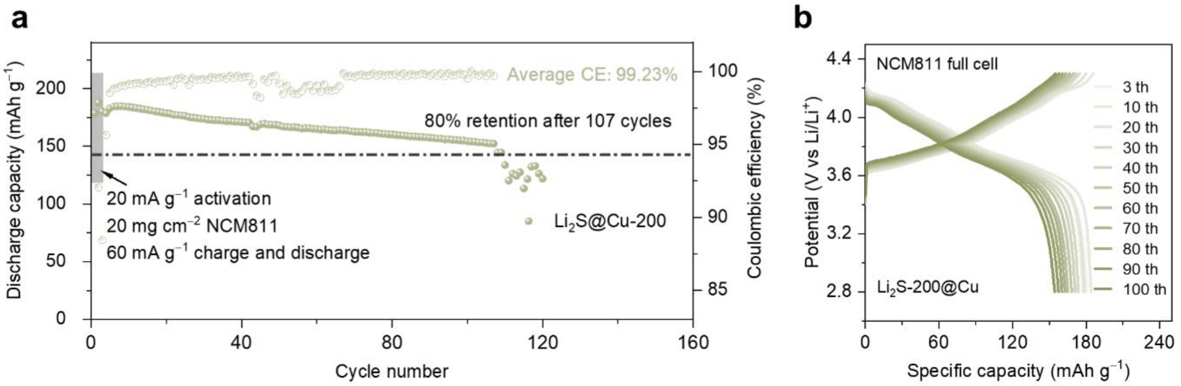

Supplementary Fig. 66| The electrochemical performance of the NCM811 full cell based on Li_2S-200@Cu substrate. (a) Discharge capacity retention of Li-substrate||NCM811 full cells at 60 mA g^{-1} (1 C = 200 mA g^{-1}). (b) Corresponding galvanostatic charge/discharge profiles of NCM811 full cells at different cycles.

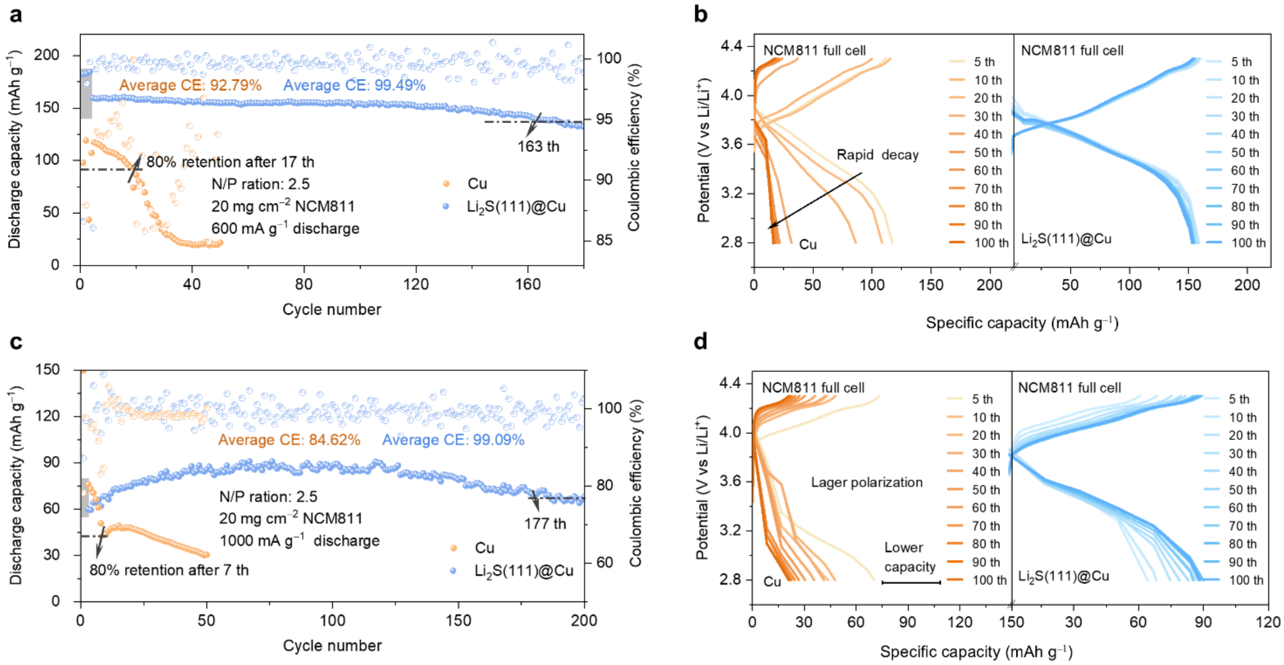

Supplementary Fig. 67| Electrochemical performance of NCM811 full cells based on Cu and Li_2S(111)@Cu substrates at high rates. (a) Discharge capacity retention of Li-substrate||NCM811 full cells at 600 mA g^{-1} discharge and 66 mA g^{-1} charge. (b) Corresponding GDC profiles of NCM811 full cells in (a) at various cycles . (c) Discharge capacity retention of Li-substrate||NCM811 full cells at 1000 mA g^{-1} discharge and 100 mA g^{-1} charge. (d) Corresponding GDC profiles of NCM811 full cells in (c) at various cycles. 1 C = 200 mA g^{-1}.

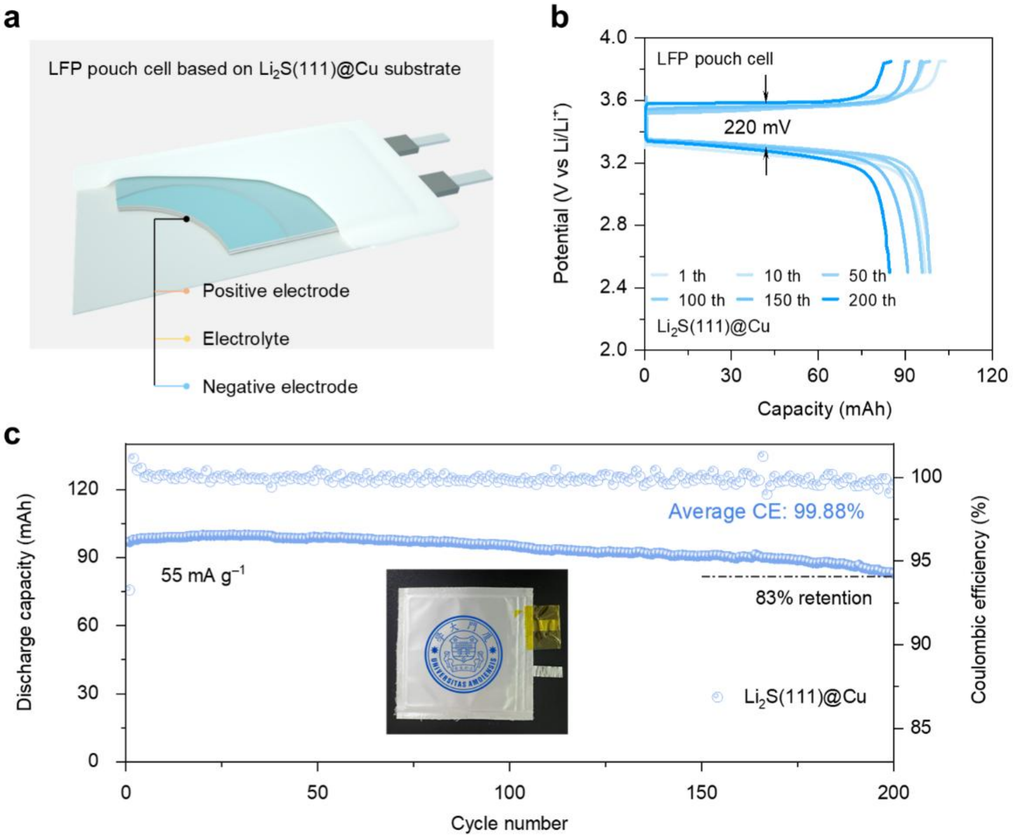

Supplementary Fig. 68| Electrochemical performance of LFP pouch cell based on the $Li_2S(111)$@Cu substrate. (a) Schematic illustration of the LFP pouch cell. (b) Corresponding GDC profiles of the LFP pouch cell at different cycles. (c) Cycling performance of the LFP pouch cell at 55 mA g^{-1} (1 C = 167 mA g^{-1}).

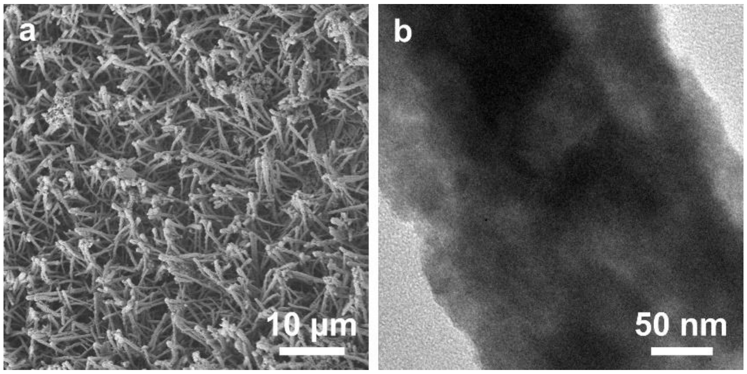

Supplementary Fig. 69| The morphology of the Li_2S(111)@Cu substrate after 100 cycles. (a) SEM image. (b) TEM image.

Supplementary Table 1. Summary of the calculated binding energy and charge transfer results of Li adsorbed at different sites on Li_2S(311).

Position	Binding (eV)	Population (e^-)
A	-0.6277	0.2452
B1	-1.4156	0.6069
B2	-1.4157	0.5892

Supplementary Table 2. Summary of the calculated binding energy and charge transfer results of Li adsorbed at different sites on $Li_2S(110)$.

Position	Binding (eV)	Population
A1	-0.3388	0.3139
A2	-0.3298	0.2784
A3	-0.3379	0.3044
B	-0.3419	0.4296
C1	-0.5833	0.2787
C2	-0.5832	0.2771
C3	-0.5833	0.2784

Supplementary Table 3. Summary of the calculated binding energy and charge transfer results of Li adsorption at different sites on $Li_2S(111)$.

Position	Binding (eV)	Population
A1	-4.2340	0.9104
A2	-4.2338	0.9103
B1	-5.1412	0.8420
B2	-5.1445	0.8422
C1	-5.3673	0.8253
C2	-5.3658	0.8246

Supplementary Table 4. EXAFS fitting parameters at the Cu K-edge for various samples (S_0^2=0.85).

Samples	Shell	CN	R(Å)	σ^2	ΔE_0	R factor
Cu foil	Cu-Cu	12	2.54±0.01	0.0086	4.7±0.5	0.0031
Cu_2S	Cu-S	4.3±0.3	2.27±0.02	0.0160	4.2±1.4	0.0185
Cu_2S_x	Cu-S	3.9±0.3	2.28±0.02	0.0170	4.9±1.7	0.0162
	Cu-Cu	0.3±0.2	2.99±0.05	0.0008		

CN: coordination numbers; R: bond distance; σ^2: Debye-Waller factors; ΔE_0: the inner potential correction.

R factor: goodness of fit.

Supplementary Table 5. Electrolyte resistance (R_e) and interfacial resistance (R_i) of Li||substrate half-cells based on various substrates before and after cycling in Figs. 4c-4d and Supplementary Fig. 51.

	Before cycling (Ω)				After cycling(Ω)			
	R_e	Error%	$R_{interfacial}$	Error%	R_e	Error%	$R_{interfacial}$	Error%
Cu	1.98	4.83	63.61	0.96	3.01	1.2	19.53	1.17
Li_2S@Cu	1.30	6.59	43.93	1.07	3.65	1.67	13.30	5.95
Li_2S(111)@Cu	2.46	3.03	16.87	3.23	2.80	1.44	6.48	1.53

Supplementary Table 6. The calculated ionic conductivity from the Nyquist plots of different substrates.

	Ionic conductivity ($S\ cm^{-1}$)
Cu	1.5×10^{-2}
Li_2S@Cu	1.6×10^{-2}
Li_2S(111)@Cu	2.2×10^{-2}

Supplementary Table 7. Comparison of electrochemical performance of full cells employing different substrates reported in literatures and this work. LFP: 1 C = 167 mA g^{-1}, NCM811: 1 C = 200 mA g^{-1}.

Materials	Electrolytes	N/P ratio	Positive electrode areal capacity	Current density (mA g^{-1})	Lifespan	Ref
3D Cu-Zn substrate	1 M LiTFSI in DOL/DME with 1% $LiNO_3$	3.3	0.3 mAh cm^{-2} LFP	83.5	300 cycles	1
Nanowires Cu_2S–Cu substrate	1 M LiTFSI in DOL/DME with 1% $LiNO_3$	2	1 mAh cm^{-2} LFP	83.5	320 cycles	2
3D Cu@PDMS substrate	1 M LiTFSI in DOL/DME with 1% $LiNO_3$	3	1 mAh cm^{-2} LFP	167	100	3
Ni-TAA/Cu substrate	1 M $LiPF_6$ in EC/DEC (1/1)	5.9	0.85 mAh cm^{-2} LFP	167	400 cycles	4
CuO@Cu substrate	1 M LiTFSI in DOL/DME with 1% $LiNO_3$	6	0.5 mAh cm^{-2} LFP	167	300 cycles	5
Sheet-like Cu@Li_2S substrate	1 M LiTFSI in DOL/DME with 1% $LiNO_3$	3.3	1.8 mAh cm^{-2} LFP	167	200 cycles	6
Nanowire CuS substrate	1 M LiTFSI in DOL/DME with 2% $LiNO_3$	7	N/A mAh cm^{-2} LFP	167	200 cycles	7
Co-2@NF substrate	1 M LiTFSI in DOL/DME with 1% $LiNO_3$	14.1	0.42 mAh cm^{-2} LFP	334	480 cycles	8
CC-Ag substrate	1 M LiTFSI in DOL/DME with 1% $LiNO_3$	15	0.33 mAh cm^{-2} LFP	668	30 cycles	9
ellulose/graphene carbon composite aerogel	1 M $LiPF_6$ in 1:1 EC/DEC with 5% VC	Excess Li	1.42 mAh cm^{-2} LFP	835	1000	10
Fe-N@SSM substrate	1 M LiTFSI in DOL/DME with 2% $LiNO_3$	3.33	1.8 mAh cm^{-2} LFP	835	280 cycles	11
GP substrate	1 M $LiPF_6$ in 5:1 EMC/FEC	2	4 mAh cm^{-2} NCM532	46.5	160 cycles	12
Nanosheet Cu_2S@Cu substrate	1 M $LiPF_6$ in EC/DEC/DMC (1:1:1)	2.5	4 mAh cm^{-2} NCM811	100	180 cycles	13
SNGO substrate	1 M $LiPF_6$ in EC/DEC (1/1) with 1% VC+10% FEC	Excess Li infusion in host	1.7 mAh cm^{-2} NCM811	100	100 cycles	14

Li_2S coating	1 M $LiPF_6$ in 1:1 EC/DEC	3.6	2.8 mAh cm^{-2} NCM532	155	100 cycles	15
Fe/LiF substrate	Advanced electrolyte: 2 M LiFSI in DME/BTFE (1/4)	1	3 mAh cm^{-2} NCM811	200	130 cycles	16
Nanorods $Li_2S(111)$@Cu substrate	*1 M LiTFSI in DOL/DME with 2% $LiNO_3$*	*4*	*1 mAh cm^{-2}, LFP*	*83.5*	*400 cycles*	*This work*
		10	*1 mAh cm^{-2}, LFP*	*500*	*1150 cycles*	
		10	*1 mAh cm^{-2}, LFP*	*835*	*2060 cycles*	
	1 M $LiPF_6$ in EC/DEC with 5% FEC	*1.3*	*4 mAh cm^{-2} NCM811*	*60*	*160 cycles*	
		2.5	*4 mAh cm^{-2} NCM811*	*600*	*163 cycles*	
		2.5	*4 mAh cm^{-2} NCM811*	*1000*	*177 cycles*	

Supplementary Table 8. The mass proportion of each component the LFP pouch cell.

Cell component	Specification	Parameters	Mass proportion
LFP Positive electrode with Al substrate	Active material mass loading	20 mg cm^{-2}	12.42%
	Active material ratio	90.93%	
	Number	1	
	Weight	0.795 g	
Negative electrode (Li_2S(111)@Cu substrate with 3 mAh cm^{-2} Li)	Weight	2.022 g	31.58%
	Number	1	
Electrolyte	Weight	0.386 g	6.03%
Separator	Weight	0.045 g	0.70%
Package	Weight	2.798 g	43.70%
Tabs	Weight	0.357 g	5.57%
Total	Weight	6.403 g	100%

It should be noted that the high percentage of the negative electrode in the total mass of the full cell is mainly due to the heavy weight of the Cu foam used during the synthesis, and the mass of Li_2S(111)@Cu is only 6.67% heavier than Cu foam. Thus, we believe that by replacing the Cu foam with lighter Cu foil, the Li_2S(111)@Cu foil substrate could be commercially viable.

Supplementary Note 1. Li conformations at different sites and diffusion pathways.

In Supplementary Fig. 2, the Li adsorption $E_{binding}$ at A1, A2 and A3 sites on $Li_2S(110)$ is essentially identical because these three sites are equivalent (denoted as A site). The $E_{binding}$ value for A site in Fig.1b represents the average of A1, A2 and A3. Similarly, the C1, C2 and C3 sites constitute a single type of adsorption site (denoted as C site). Similarly, the $E_{binding}$ values for the A, B and C sites on $Li_2S(111)$ in Fig. 1c represent the averages of A1 and A2, B1 and B2, as well as C1 and C2, respectively, as shown in Supplementary Fig. 3. The diffusion pathways are determined based on the hopping of Li from the most stable to less stable position, ie, from B1 to B2 in Fig. 1g, and C1 to C2 in Fig. 1h and 1i.

Supplementary Note 2. Defect effects

It has been reported that the presence of defects can disrupt the periodic crystalline structure of materials, and then alter the surface electronic structure with localized electron re-distribution[17,18]. During the reaction between Cu_2S and Li, the S sublattice rearranged as Cu left the structure, and the close-packed S planes shifting from the hcp-type stacking of Cu_2S to the fcc-type stacking of Li_2S[19]. In the high chalcocite Cu_2S, the crystallography of the structure is defined by the hexagonal sulfur framework (space group $Fm\bar{3}m$)[20]. Various in S defect content may alter the arrangement of S sublattice, thus potentially influencing the crystal orientation of Li_2S. On the other hand, after inducing S defects in Cu_2S@Cu, the electron density around Cu atoms increased, as indicated by the lower Cu binding energies and higher S binding energies in XPS spectra of Cu_2S_x@Cu. Specifically, the *Cu* $2p_{1/2}$ and $2p_{3/2}$ peaks of Cu_2S@Cu are located at 954.1 and 934.3 eV, respectively, while the *S* $2p_{1/2}$ and $2p_{3/2}$ peaks are at 161.4 and 162.6 eV, respectively. In the case of Cu_2S_x@Cu, the *Cu* $2p_{1/2}$ and $2p_{3/2}$ peaks are located at 952.1 and 932.3 eV, and the *S* $2p_{1/2}$ and $2p_{3/2}$ peaks are recorded at 161.5 and 162.7 eV, respectively (Supplementary Fig. 5). The increased EPR signal also reflected more unpaired electron in Cu_2S_x than Cu_2S (Fig. 2b). The XAS results revealed a lower valence state of Cu and a decreased coordination number in Cu_2S_x than in Cu_2S (Supplementary Figs. 7-8). In Supplementary Fig. 7a, the similar near-edge features observed in the Cu K-edge spectra of both Cu_2S and Cu_2S_x suggest the preserved crystal structure after the annealing process. The valence state of Cu in Cu_2S_x is found to locate between +1 and 0. The peak at around 2.2 Å represents the Cu-Cu bond in Cu foil. The main peak of Cu_2S and Cu_2S_x at ~1.9 Å corresponds to the first shell scattering of the Cu-S bond, and the lower intensity attenuation in Cu_2S_x indicates a decreased coordination of Cu and the introduction of sulfur defects. In Supplementary Fig. 8, the wavelet transform (WT) analysis revealed a distinct intensity peak in both samples, attributed to the formation of Cu-Cu bond in Cu foil, and a Cu-S bond in Cu_2S and Cu_2S_x. The shift of radial distance in a negative direction for the Cu-S bond compared to Cu-Cu is consistent with Supplementary Fig. 7b. The color variation in the Cu-S coordination zone is associated with the differing coordination environment between Cu_2S and Cu_2S_x.

The introduction of defects can also regulate the conductivity of materials, influencing the density and

activity of active sites[17]. In this work, the sulfur defect of Cu_2S_x (20.9 Ω) led to a higher conductivity compared to Cu_2S (51.3 Ω), which directly impacts the electron-transfer rate during the Li activation process. The intermediate phase in the Cu_2S_x system appeared earlier and persisted longer than in the Cu_2S system (Supplementary Figs. 15 and 18). In addition, the surface of Cu_2S_x NRs exhibited increased roughness than Cu_2S NRs, as observed in the TEM images in Supplementary Figs. 10-11, resulting in an increased number of active sites during lithiation process. The online differential electrochemical mass spectrometry (OEMS) tests showed obvious signals of CO_2 and C_2H_4 gas during the lithiation process in Cu_2S system (see Supplementary Fig. 26). However, no gas evolution occurred in the Cu_2S_x system, indicating a distinct electron transfer mechanism from the electrode to the electrolyte compared to the Cu_2S system. In summary, the introduction of defects could alter the sublattice arrangement of Cu_2S_x, and regulate the electronic structure of material, thus providing a means to control the Li activation process in Cu_2S_x system. This finding holds potential significance in the construction of (111) faceted Li_2S.

Supplementary Note 3. Lattice expansion

In Supplementary Fig. 4b, the XRD characteristic peaks of Cu_2S(102), (110) and (103) facets are located at 37.67°, 46.15° and 48.66°, respectively. After introducing sulfur defects, these three peaks undergo a shift to 37.35°, 45.89° and 48.31°, respectively. It should be noted that the lower 2θ values in Cu_2S_x@Cu sample are due to the influence of lattice expansion[21]. The removal of sulfur gives rise to coordinatively unsaturated metal centers, resulting in electrostatic repulsion between positively charged sulfur defects and the surrounding copper cations, and the subsequent lattice expansion[22].

Supplementary Note 4. In situ XRD analysis of the Li_2S@Cu formation process

In Supplementary Fig. 17, during the initial stage of Li_2S@Cu formation process, we observe behaviors like those mentioned earlier, such as twice-negative peak shifts and the formation of intermediate phases. However, the first negative shift of Cu_2S diffraction peaks occur at a discharge depth of 7% (SOD 7%, where defined discharge to 0 V as SOD 100%), This shift happens later than that of Cu_2S_x (SOD 3%), and the second peak shifts at SOD 28%, which is earlier than that of Cu_2S_x (SOD 31%). Following the second peak shift, the characteristic peaks of Li_2S(111), (220), (311) and (222) facets are detected at 27.1°, 45.1°, 53.4° and 55.8°, respectively. The eventual substrate obtained after Li activation of Cu_2S@Cu is denoted as Li_2S@Cu.

Supplementary Note 5. In situ XRD analysis of the Li_2S-200@Cu formation process

In Supplementary Fig. 22, the Cu_2S-200@Cu exhibited similar behaviors like the Cu_2S@Cu and the Cu_2S_x@Cu during the initial Li activation process, such as the peak shifts and the formation of intermediate phase. The two negative shift of Cu_2S-200 diffraction peaks occur at SOD 4% and SOD 28%, respectively. The characteristic peaks of Li_2S(111), (220), (311) and (222) facets are detected at 27.0°, 45.5°, 53.2° and 55.9°, respectively. The eventual substrate obtained after Li activation of Cu_2S-200@Cu is denoted as Li_2S-200@Cu.

Supplementary Note 6. The XRD peak intensity variations of the detected signals of Cu(111) and Cu(200)

In Supplementary Fig. 23, we summarized the peak intensity variations of the detected signals of Cu(111) and Cu(200) during the in-situ electrochemical lithiation of Cu_2S@Cu and Cu_2S_x@Cu. Both of the intensities of Cu(111) and Cu(200) underwent an initial increase, followed by stabilization as the lithiation progresses. Meanwhile, the intensity of Cu(111) remained higher than Cu(200) throughout, suggesting the Cu clusters in Li_2S@Cu and Li_2S(111)@Cu substrates exhibited the (111) dominated surface as the native Cu. Supplementary Fig. 22c displayed the variations of the peak intensity ratio of Cu(111)/Cu(200), the value in Cu_2S@Cu and Cu_2S_x@Cu were 1.67 and 1.81, respectively; after Li activation process, the value in Li_2S@Cu and Li_2S(111)@Cu increased to 2.08 and 2.31, respectively. These changes suggested that the increasement of the proportion of (111) facets in the Cu clusters. Overall, the Cu clusters formed through the in-situ electrochemical lithiation of Cu_2S and Cu_2S_x both exhibited the (111) dominated surface, as well as the native Cu substrate. Therefore, the variations in battery performance were mainly attributed to the different crystal facets of the generated Li_2S.

Supplementary Note 7. Morphological analysis of Li deposited on the Li_2S(111)@Cu substrate

Supplementary Figs. 28a and 28d show the surface of the initial Li_2S(111)@Cu NRs is smooth. Compared with the initial Li_2S(111)@Cu substrate (Supplementary Fig. 28d), the luminous white spots appear after depositing Li (Supplementary Fig. 29a). The corresponding gray value intensity distribution shows a noticeable difference in gray levels within these luminous white spots, which are the deposited Li particles (Supplementary Fig. 29b). After a slight amount of Li deposition, such as 0.001 and 0.002 mAh cm^{-2} in Supplementary Figs. 28b-28f, some luminous white spots appear on the surface of nanorods, which are considered to be the deposited Li particles. The initial Li particles exhibit a spherical shape (Supplementary Fig. 28e, 0.001 mAh cm^{-2}), and then aggregate into droplet-shaped particles (Supplementary Fig. 28f, 0.002 mAh cm^{-2}).

Supplementary Note 8. Morphological analysis of Li deposited on the Li_2S@Cu substrate

During the initial stage of Li deposition (0.001 and 0.002 mAh cm^{-2}), minute Li particles adhere on the Li_2S@Cu substrate's surface in a point-contact manner (Supplementary Figs. 30e-30f). This mode of adhesion is different from the surface-contact manner observed on the Li_2S(111)@Cu NRs surface (Fig. 3f-3g). When the deposition capacity reaches 1 mAh cm^{-2}, Li fails to fully envelop the Li_2S@Cu NRs (Supplementary Fig. 31a). Upon further Li plating, reaching a capacity of 10 mAh cm^{-2}, a loosely Li deposited layer is formed on the top surface of Li_2S@Cu NRs substrates (Supplementary Fig. 31b), and the deposition of Li tends to follow the "Stranski-Krastanov" growth mode[23]. Despite the irregular crystal orientation of Li_2S@Cu NRs surfaces, which strongly affects the texture of Li compared to the dendritic growth of Li on Cu substrate, the 3D vertical nanorods array and chemical feature of Li_2S still confer the advantage of inducing a much flatter Li deposition.

Supplementary Note 9. Morphological analysis of Li deposited on different substrates after first cycle

After the first cycle, the 3D vertical array structure of the $Li_2S(111)$@Cu substrate remained and almost no inactive Li could be observed (Supplementary Figs. 32a and 32d). When a trace amount of Li deposited on the cycled $Li_2S(111)$@Cu substrate, the sphere-shaped Li particles appeared (Supplementary Figs. 32b and 32e, 0.001 mAh cm^{-2}), and then aggregated (Supplementary Figs. 32c and 32f, 0.002 mAh cm^{-2}) as the behavior on the fresh $Li_2S(111)$@Cu substrate (Supplementary Fig. 24). However, limited inactive Li remained on the Li_2S@Cu substrate after the first cycle (Supplementary Fig. 33a). After plating 0.001 and 0.002 mAh cm^{-2} Li, the Li particles were adhered on the cycled Li_2S@Cu substrate in a point-contact configuration (Supplementary Figs. 33b-33f), which was similar to the manner observed on the fresh Li_2S@Cu substrate. For the Cu substrate, the SEM image in Supplementary Fig. 34a showed clear inactive Li after the first cycle. After plating 0.001 and 0.002 mAh cm^{-2} Li, the irregularly deposited Li particles quickly aggregated to form dendrites (Supplementary Figs. 34b-34c). As the plating Li amount increased to 1 and 10 mAh cm^{-2}, the deposited Li could entirely cover the cycled $Li_2S(111)$@Cu nanorods and eventually form a dense Li layer (Supplementary Figs. 35a and 35d). Whilst with the cycled Li_2S@Cu substrate, the plated Li did not fully envelop the nanorods (Supplementary Fig. 35b) due to the lack of regularity in the crystal orientation, resulting in a loose Li deposited layer (Supplementary Fig. 35e). For the cycled Cu substrate, after plating 1 and 10 mAh cm^{-2} Li, substantially Li dendrites were presented on the surface (Supplementary Figs. 35c and 35f).

Supplementary Note 10. Morphological analysis of Li deposited on the Li_2S-200@Cu substrate

During the initial stage of Li deposition, such as deposited 0.001 and 0.002 mAh cm^{-2} Li (Supplementary Figs. 36b and 36e, 36c and 36f), two manners of deposition were observed on the Li_2S-200@Cu substrate's surface. Specifically, some of Li particles adhered on the Li_2S-200@Cu NRs surface in a surface-contact manner, as marked in gold dashed circles in (Supplementary Figs. 35e-36f), this mode is similar with that of manner observed on the Li_2S(111)@Cu NRs surface (Supplementary Fig. 28). Meanwhile, part of Li particles displayed a point-contact manner (highlighted by red dashed circles), as that of mode observed on the Li_2S@Cu NRs surface (Supplementary Fig. 30). When the deposition amount increased to 1 mAh cm^{-2}, the aggregated Li displayed a seaweed-like clusters morphology, distributed in the Li_2S-200@Cu NRs array, showing a shaggy structure (Supplementary Fig. 37a). This is different from the completely enveloped formed in the Li_2S(111)@Cu NRs by deposited Li (Fig. 4h). As the Li deposition amount increase, SEM image as shown in (Supplementary Fig. 37b) reveal a partially dense Li layer on the top surface Li_2S-200@Cu NRs substrate.

Supplementary Note 11. Scharifker equations

Based on the Schariker and Hills (S-H) theory, the mathematical expressions of the classical instantaneous and progressive nucleation theory are presented by the following Scharifker equation (1) and (2), respectively[24,25]:

$$\frac{I^2}{I_m^2} = \frac{1.9542}{t/t_m}\left\{1 - exp\left[-1.2564\left(t/t_m\right)\right]\right\}^2 \qquad (1)$$

$$\frac{I^2}{I_m^2} = \frac{1.2254}{t/t_m}\left\{1 - exp\left[-2.3367\left(t/t_m\right)^2\right]\right\}^2 \qquad (2)$$

where I_m and t_m can be determined by equating the first derivative of the I-t curves to zero.

Supplementary Note 12. Analysis of the high-rate performance of LFP full cells

In Supplementary Fig. 63, the LFP full cell based on the $Li_2S(111)$@Cu substrate displayed a steady capacity at a high current density of 500 mA g^{-1}, with capacity retentions of 80% after 1150 cycles, and 75% after 1500 cycles. However, the full cell based on Cu substrate exhibited a pronounced deterioration only after 54 cycles. Incidentally, the capacity fluctuations observed in later cycles may result from the variations in internal properties during long-term cycling as well as fluctuations in the ambient lab temperature[26]. Even at a high current density of 835 mA g^{-1}, the LFP full cell based on the $Li_2S(111)$@Cu substrate still maintained high stability for 2060 cycles with a capacity retentions of 80%, and maintained 73% capacity after 2500 cycles. Meanwhile, the LFP full cell based on Cu substrate still experienced rapid degradation, and the average CE was as low as 69.0% during the first 50 cycles, followed by significant fluctuations.

Supplementary References

1. Yun, Q. et al. Chemical dealloying derived 3D porous current collector for Li metal anodes. *Adv. Mater.* **28**, 6932-6939 (2016).
2. Zhai, P. et al. In situ generation of artificial solid-electrolyte interphases on 3D conducting scaffolds for high-performance lithium-metal anodes. *Adv. Energy Mater.* **10**, 1903339 (2020).
3. Wang, X. et al. Stress-driven lithium dendrite growth mechanism and dendrite mitigation by electroplating on soft substrates. *Nat. Energy.* **3**, 227-235 (2018).
4. Ke, S.-W. et al. Redox-active covalent organic frameworks with nickel−bis(dithiolene) units as guiding layers for high-performance lithium metal batteries. *J. Am. Chem. Soc.* **144**, 8267-8277 (2022).
5. Liu, Y. et al. Integrated gradient Cu current collector enables bottom-up Li growth for Li metal anodes: role of interfacial structure. *Adv. Sci (Weinh).* **10**, 2301288-2301295 (2023).
6. Zhang, R. et al. Decreasing interfacial pitfalls with self-grown sheet-like Li_2S artificial solid-electrolyte interphase for enhanced cycling performance of lithium metal anode. *Small.* **19**, 2208095 (2023).
7. Zou, P., Wang, C., Qin, J., Zhang, R. & Xin, H. L. A reactive wetting strategy improves lithium metal reversibility. *Energy Stor. Mater.* **58**, 176-183 (2023).
8. Guo, C. et al. Uniform lithiophilic layers in 3D current collectors enable ultrastable solid electrolyte interphase for high-performance lithium metal batteries. *Nano Energy.* **96**, 107121 (2022).
9. Tian, R. et al. Oriented growth of Li metal for stable Li/carbon composite negative electrode. *Electrochim. Acta.* **292**, 227-233 (2018).
10. Mao, H. et al. Current-density regulating lithium metal directional deposition for long cycle-life Li metal batteries. *Angew. Chem. Int. Ed.* **60**, 19306-19313 (2021).
11. Fu, X., Duan, H., Zhang, L., Hu, Y. and Deng, Y. A 3D framework with an in situ generated Li_3N solid electrolyte interphase for superior lithium metal batteries. *Adv. Funct. Mater.* **33**, 2308022 (2023).
12. Li, Y. et al. Artificial graphite paper as a corrosion-resistant current collector for long-life lithium metal batteries. *Adv. Funct. Mater.* **33**, 2214523 (2023).
13. Yang, Z. et al. Ultra-smooth and dense lithium deposition toward high-performance lithium metal

batteries. *Adv. Mater* **35**, 2210130 (2023).

14. Ni, S. et al. A 3D framework with Li_3N-Li_2S solid electrolyte interphase and fast ion transfer channels for a stabilized lithium-metal anode. *Adv. Mater* **35**, 2209028 (2023).
15. Chen, H. et al. Uniform high ionic conducting lithium sulfide protection layer for stable lithium metal anode. *Adv. Energy Mater* **9**, 1900858 (2019).
16. Wu, Z. et al. Growing single-crystalline seeds on lithiophobic substrates to enable fast-charging lithium-metal batteries. *Nat. Energy* **8**, 340-350 (2023).
17. Xie, C. et al. Insight into the design of defect electrocatalysts: From electronic structure to adsorption energy. *Mater. Today.* **31**, 47-68 (2019).
18. Zhang, S. et al. Configuration regulation of active sites by accurate doping inducing self-adapting defect for enhanced photocatalytic applications: A review. *Chem. Rev.* **478**, 214970 (2023).
19. McDowell, M. T. et al. In situ observation of divergent phase transformations in individual sulfide nanocrystals. *Nano Lett.* **15**, 1264-1271 (2015).
20. Buerger, M. J., Bernhardt, J. W. Distribution of atoms in high chalcocite, Cu_2S. *Science.* **141**, 276-277 (1963).
21. Liu, X. et al. Uncovering the effect of lattice strain and oxygen deficiency on electrocatalytic activity of perovskite cobaltite thin films. *Adv. Sci. (Weinh)* **6**, 1801898 (2019).
22. Qian, K. et al. Elucidating the strain-vacancy-activity relationship on structurally deformed Co@CoO nanosheets for aqueous phase reforming of formaldehyde. *Small*. **17**, 2102970 (2021).
23. Budevski, E. Staikov, G. Lorenz, W.J. *Electrochemical Phase Formation and Growth* (VCH Verlagsgesellschaft mbH, Weinheim. Press, 1996).
24. Benjamin, S. Graham, H. Heoretical and experimental multiple nucleation. *Electrochim. Acta.* **28**, 879-889 (1983).
25. Thirumalraj, B. et al. Nucleation and Growth Mechanism of Lithium Metal Electroplating. *J. Am. Chem. Soc.* **141**, 18612-18623 (2019).
26. Krieger, E. M., Cannarella, J. and Arnold, C. B. A comparison of lead-acid and lithium-based battery behavior and capacity fade in off-grid renewable charging applications. *Energy.* **60**, 492-500 (2013).
