# Supplementary material for: Sulfur defect engineering controls Li2S crystal orientation towards dendrite-free lithium metal batteries
Source: Nat Commun. 2025 Apr 1;16:3130. doi: 10.1038/s41467-025-57572-5 (PMC11962132; doi:10.1038/s41467-025-57572-5)
Supplement: Supplementary file 3 — Description of Additional Supplementary Files [file 41467_2025_57572_MOESM3_ESM.pdf]

Description of Additional Supplementary Files

Supplementary Data legends

File Name: Supplementary Data 1
Legend: The optimized computational model of the $Li_2S(311)$ geometrical structure.

File Name: Supplementary Data 2
Legend: The optimized computational model of the $Li_2S(110)$ geometrical structure.

File Name: Supplementary Data 3
Legend: The optimized computational model of the $Li_2S(111)$ geometrical structure.

File Name: Supplementary Data 4
Legend: The optimized computational model of the charge density difference of Li adatom on $Li_2S(311)$.

File Name: Supplementary Data 5
Legend: The optimized computational model of the charge density difference of Li adatom on $Li_2S(110)$.

File Name: Supplementary Data 6
Legend: The optimized computational model of the charge density difference of Li adatom on $Li_2S(111)$.

Supplementary Movie legends

Supplementary Movie 1. In situ optical microscopy video of Li deposited on $Li_2S(111)$@Cu NRs substrate.

Supplementary Movie 2. In situ optical microscopy video of Li deposited on Cu substrate.

Supplementary Movie 3. In situ optical microscopy video of Li deposited on Li_2S@Cu NRs.
